# From Cellular Radiosensitivity to Precision Radiotherapy: Integrating Functional Assays, Genomics, and Clinical Modeling

**DOI:** 10.3390/cancers18111823

**Published:** 2026-06-02

**Authors:** Angeliki Gkikoudi, Sotiria Triantopoulou, Eygenia Markellou, Vasiliki Xynou, Spyridon N. Vasilopoulos, Marios Myronakis, Evagelia C. Laiakis, Kiki Theodorou, Georgia I. Terzoudi, Alexandros G. Georgakilas

**Affiliations:** 1 DNA Damage Laboratory, Physics Department, School of Applied Mathematical and Physical Sciences, National Technical University of Athens (NTUA), Zografou Campus, 15780 Athens, Greece; angelikigkikoudi@mail.ntua.gr (A.G.); eygeniamarkellou891@gmail.com (E.M.); svasilopoulos@mail.ntua.gr (S.N.V.); 2 Laboratory of Health Physics, Radiobiology & Cytogenetics, Institute of Nuclear & Radiological Sciences & Technology, Energy & Safety, National Centre for Scientific Research “Demokritos”, 15341 Agia Paraskevi, Greece; iro@rrp.demokritos.gr (S.T.); vasiliki.ksn@gmail.com (V.X.); gterzoudi@rrp.demokritos.gr (G.I.T.); 3 Department of Medical Physics, Faculty of Medicine, University of Thessaly, 41500 Larissa, Greece; myronakis@uth.gr (M.M.); ktheodor@med.uth.gr (K.T.); 4 Department of Radiation Medicine, Georgetown University Medical Center, Washington, DC 20057, USA; ecl28@georgetown.edu; 5 Department of Oncology, Lombardi Comprehensive Cancer Center, Georgetown University Medical Center, Washington, DC 20057, USA; 6 Department of Biochemistry and Molecular & Cellular Biology, Georgetown University Medical Center, Washington, DC 20057, USA; 7 Innovation and Research, King Faisal Specialist Hospital & Research Centre, Riyadh 11211, Saudi Arabia

**Keywords:** radiotherapy toxicity, radiosensitivity, functional radiosensitivity assays, DNA damage response, γ-H2AX, RILA, chromosomal radiosensitivity, micronucleus assay, organoids, personalized radiotherapy

## Abstract

**Background**: Radiotherapy outcomes are determined by the balance between tumor control and normal tissue toxicity, both of which exhibit significant interpatient variability. While radiogenomic and molecular approaches have identified determinants of radiosensitivity, their predictive performance remains limited when used in isolation. **Methods**: This review provides a comprehensive synthesis of functional assays, genomic biomarkers, and integrative models used to assess radiosensitivity, including radiation-induced lymphocyte apoptosis (RILA), chromosomal radiosensitivity assays, micronucleus formation, γ-H2AX foci kinetics, comet assays, clonogenic survival, and patient-derived organoids, alongside genomic and molecular predictors of tumor response such as hypoxia signatures and gene expression-based models (e.g., RSI and GARD). **Results**: Functional assays provide direct phenotypic assessment of radiation response, capturing DNA repair capacity, chromosomal stability, apoptosis, and tissue regenerative potential, and have shown associations in several studies with normal tissue toxicity across clinical settings. Tumor radiosensitivity is influenced by intrinsic cellular factors and microenvironmental conditions, including hypoxia and genomic instability. Integrative approaches combining functional, genomic, and clinical data show increasing potential for improving predictive accuracy. **Conclusions**: Radiosensitivity should be considered a systems-level phenotype requiring multi-dimensional assessment. Conceptual frameworks that integrate tumor and normal tissue responses within a unified modeling approach, supported by measurable biological and clinical parameters, represent a promising direction for clinically actionable precision radiotherapy. The integration of functional assays with genomic and clinical modeling frameworks represents a promising strategy for advancing personalized radiotherapy and improving therapeutic outcomes.

## Simple Summary

Radiotherapy response varies among patients due to differences in tumor and normal tissue radiosensitivity. This review outlines key biological factors influencing this variability, including DNA repair capacity, genetic background, and the tumor microenvironment. It summarizes functional assays used to assess radiation response and highlights genomic approaches such as gene expression models and hypoxia-related markers. The importance of integrating biological, molecular, and clinical data is emphasized to improve prediction of treatment outcomes.

## 1. Introduction

Radiotherapy operates within a narrow therapeutic window defined by two biologically distinct yet clinically interconnected endpoints: normal tissue toxicity and tumor control. Clinically, radiotherapy response reflects the balance between tumor control probability (TCP) and normal tissue complication probability (NTCP), both of which arise from shared but context-dependent biological processes. While both outcomes arise from radiation-induced DNA damage and downstream cellular responses, they are governed by different biological determinants and require distinct, though sometimes overlapping, biomarker strategies. Interpatient variability in normal tissue toxicity remains one of the most persistent challenges in radiotherapy. Patients receiving comparable dose–volume exposures frequently exhibit markedly different acute and late adverse effects, including fibrosis, organ dysfunction, mucosal injury, skin toxicities and long-term impairment in quality of life [1,2]. This variability reflects intrinsic biological differences collectively referred to as normal tissue radiosensitivity. Normal tissue radiosensitivity is shaped by variation in DNA damage response (DDR) efficiency, double-strand break (DSB) repair fidelity, checkpoint activation, apoptotic competence, inflammatory signaling, vascular response, and epithelial stem cell regenerative capacity [3,4]. Importantly, germline genetic variation contributes substantially to this phenotype. Single nucleotide polymorphisms (SNPs) and rare variants in genes involved in DDR (e.g., ataxia–telangiectasia mutated (*ATM*), breast cancer susceptibility gene 1/2 (*BRCA1/2*); X-ray repair cross-complementing protein 1 (*XRCC1*)), which regulate DNA damage sensing, signaling, and repair of DSBs; oxidative stress pathways, which control cellular redox balance and reactive oxygen species (ROS) detoxification; and profibrotic signaling (e.g., *TGFB1*), which mediates radiation-induced inflammation, tissue remodeling, and fibrosis, have been associated with differential toxicity risks [5,6,7,8]. Notably, even heterozygous variants in key DDR genes may influence radiosensitivity through haploinsufficiency, resulting in reduced DNA repair capacity and altered cellular responses to radiation. However, individual polymorphisms typically confer modest effect sizes, reflecting the polygenic and context-dependent nature of radiosensitivity [9,10]. Functional radiosensitivity assays have therefore been developed to capture the downstream biological consequences of this inherited variability. Platforms such as radiation-induced lymphocyte apoptosis (RILA), G2 chromosomal radiosensitivity testing, micronucleus formation, γ-H2AX foci kinetics, comet assays, clonogenic survival, and patient-derived normal tissue organoids directly interrogate radiation-induced cellular responses in patient-derived material [11,12,13,14,15,16,17,18]. These assays demonstrate mechanistically consistent associations with acute and late toxicity across multiple disease sites rather than measuring genetic predisposition alone.

Tumor control, in contrast, is determined by a more complex, multilevel interplay of tumor-intrinsic and host-related factors. While intrinsic tumor radiosensitivity, driven by DNA repair capacity, cell-cycle dynamics, hypoxia, clonal heterogeneity, and oncogenic mutations, remains central, it does not fully account for clinical outcomes [19]. Tumor control probability is influenced by:

- Tumor-intrinsic biology: DDR alterations, genomic instability, proliferative kinetics, hypoxia, cancer stem-cell burden.
- Host (patient) factors: immune competence and recruitment, systemic inflammatory profile, vascular integrity, treatment tolerance, and the microbiome, which is increasingly recognized as a modulator of radiotherapy response and toxicity through its effects on immune regulation, inflammation, and metabolic signaling, although its precise role remains incompletely understood [20].
- Germline genetic polymorphisms: inherited variation in DDR, immune-regulatory, and inflammatory genes may influence not only toxicity susceptibility but also tumor response.
- Treatment delivery factors: interruptions, dose reductions, fractionation modifications secondary to toxicity, as well as treatment modality and protocol (e.g., hypofractionation or particle therapy), which shape dose delivery, linear energy transfer, and downstream biological effects on both tumor and normal tissues [21,22,23,24].

**Figure 1:**
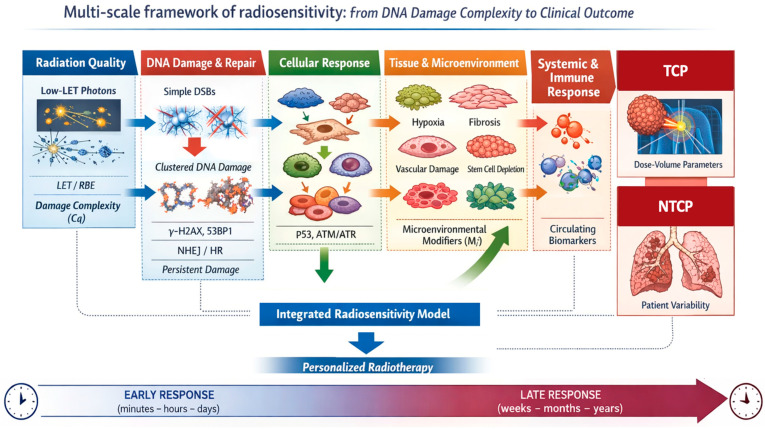
Multi-scale determinants of radiotherapy response. The different arrow colors represent transitions between distinct biological scales/processes within the integrated radiosensitivity framework and are used for visual guidance rather than quantitative weighting.

Many molecular pathways underpinning toxicity and tumor response overlap, particularly those governing DNA damage sensing, repair, apoptosis, and chromosomal stability. Consequently, certain functional platforms, such as γ-H2AX kinetics, clonogenic survival assays, and organoid systems, can be applied to both normal and tumor tissues. However, interpretation must remain context-specific. When assessed in normal tissue, these assays inform toxicity risk and when assessed in tumor cells or tumor-derived organoids, they inform tumor radiosensitivity and control probability. Nevertheless, the ability of these approaches to predict long-term clinical outcomes, including late normal tissue toxicity and tumor recurrence, remains limited, and robust molecular predictive criteria for these endpoints are still lacking. Failure to distinguish these applications risks conflating biological interpretation and clinical intent. By distinguishing normal tissue radiosensitivity from tumor control, while acknowledging their biological interdependence, this review examines the functional assay platforms developed primarily for toxicity prediction and contextualizes their relevance within the broader framework of integrated, personalized radiotherapy. Bioinformatic analyses have identified key determinants including ATM/ATR signaling, homologous recombination and non-homologous end joining components, p53 pathway integrity, and redox-modulating genes as central modulators of interindividual and tumor-specific radiation response [25]. Mechanistically, radiation-induced DSBs, replication stress, mitochondrial ROS amplification, and downstream cell fate decisions such as apoptosis, senescence, or mitotic catastrophe further shape treatment outcomes [26]. However, additional radiation-responsive pathways, including ferroptosis, autophagy, mitophagy, and regulated necrosis, are increasingly recognized but remain incompletely characterized in the context of radiotherapy response and toxicity [27]. Collectively, these findings support the view that radiosensitivity represents a system-level phenotype integrating genomic instability, stress signaling, and immune activation rather than a single molecular defect. Among currently available assays, only RILA presently approaches the level of prospective multicenter validation required for potential clinical translation, particularly for the prediction of late normal tissue toxicity. In contrast, several other assays remain primarily mechanistic or exploratory platforms with more heterogeneous reproducibility and more limited prospective validation. Radiosensitivity should be interpreted as a multi-dimensional, time-dependent phenotype that cannot be captured by single biomarkers but requires integration of functional, genomic, and clinical determinants. This review is structured in three parts. First, we examine the functional assays used to assess individual radiosensitivity, particularly in the context of normal tissue toxicity. Second, we review tumor-specific biomarkers and assays that aim to predict radiotherapy response. Finally, we discuss integrative approaches that combine functional, genomic, and clinical data to support personalized radiotherapy strategies (Figure 1). The components illustrated in Figure 1 correspond to quantifiable parameters summarized in Supplementary Table S1. The associated supplementary mathematical expressions are intended as heuristic conceptual representations of radiobiological response rather than formal TCP-NTCP prediction models. We propose that the meaningful prediction of radiotherapy outcome requires integration of functional assays, genomic biomarkers, and clinical and dosimetric parameters within a unified framework. This perspective moves beyond reductionist biomarker approaches and emphasizes the need for system-level modeling in precision radiotherapy.

Radiotherapy response is depicted as a multi-layered and time-dependent process linking physical dose delivery to patient-level outcomes. Radiation input, defined by dose, fractionation, and radiation quality (e.g., linear energy transfer (LET) and relative biological effectiveness (RBE)), induces DNA damage, including DSBs, clustered lesions, and reactive oxygen species. These lesions activate DDR pathways involving ATM/ATR signaling, checkpoint activation, and DNA repair processes such as homologous recombination and non-homologous end joining. The balance between damage induction and repair determines cellular fate decisions, including apoptosis, therapy-induced senescence, or survival and proliferation. Senescence, characterized by persistent DDR signaling and a senescence-associated secretory phenotype (SASP), exerts context-dependent effects, contributing to early tumor suppression while promoting chronic inflammation and tissue remodeling when persistent. At the tissue level, radiation-induced cellular responses influence the tumor microenvironment through immune modulation, hypoxia, stromal activation, and inflammatory signaling. These multi-scale interactions ultimately converge on clinically relevant endpoints, including TCP and NTCP. Representative biomarkers associated with each biological layer are indicated, highlighting the need for integrative, multi-dimensional approaches to predict radiotherapy response.

## 2. Functional Assays for Normal Tissue Radiosensitivity and Toxicity Risk

In clinical practice, radiotherapy is frequently delivered in combination with systemic agents, most commonly chemotherapy, which modulates radiation response at multiple biological levels. Chemoradiation therapy (CRT) enhances DNA damage through replication stress, inhibition of DNA repair pathways, and cell-cycle perturbation, thereby altering the biological interpretation of radiosensitivity biomarkers. Consequently, biomarkers validated in the context of radiotherapy alone may exhibit different predictive values when applied in combined-modality treatment settings. DNA damage response markers such as γ-H2AX and 53BP1 may reflect both increased damage induction and impaired repair under chemoradiation conditions, complicating their interpretation as indicators of intrinsic radiosensitivity. Functional assays performed in peripheral blood cells, including RILA and γ-H2AX kinetics, remain informative for normal tissue toxicity but may be influenced by systemic treatment-induced immune modulation and inflammatory signaling. Although multiple functional assays have demonstrated mechanistic associations with radiation response, their levels of clinical validation differ substantially. Some assays, particularly RILA, have been evaluated in prospective multicenter clinical cohorts and demonstrate relatively consistent predictive performance for late toxicity, whereas others remain primarily exploratory or mechanistic platforms with more limited reproducibility and clinical validation. Importantly, combined-modality treatment can also enhance therapy-induced senescence and associated inflammatory responses, contributing to both tumor control and late normal tissue toxicity. These considerations highlight the need for the context-specific interpretation of radiosensitivity biomarkers and further support the development of integrative predictive frameworks that account for treatment context and biological complexity. A comparative overview of functional radiosensitivity assays, their methodologies, and reported clinical associations is summarized in Table 1.

### 2.1. Radiation-Induced Lymphocyte Apoptosis (RILA)

RILA is an in vitro assay that measures a patient’s intrinsic sensitivity to radiation by quantifying how readily circulating CD8^+^ T lymphocytes undergo apoptosis after controlled irradiation. Because the test is performed on peripheral blood, it reflects the radiosensitivity of the patient’s normal tissues rather than the tumor. The process begins with drawing a blood sample, isolating lymphocytes, and irradiating them in vitro (commonly 8 Gy). The cells are then incubated for approximately 48 h to allow apoptotic signaling to develop. Flow cytometry is used to quantify apoptotic CD8^+^ cells, typically using Annexin V with or without propidium iodide, and the final RILA value is expressed as the percentage of apoptotic cells. Higher RILA values indicate a more robust apoptotic response to radiation-induced DNA damage, whereas lower values reflect impaired apoptotic activation [28]. The mechanistic basis of RILA is rooted primarily in p53-dependent apoptotic signaling [29]. Efficient apoptosis following irradiation may facilitate the elimination of damaged lymphocytes, thereby limiting persistent inflammatory signaling and downstream profibrotic remodeling [30]. In contrast, reduced apoptotic competence may permit the survival of genomically compromised immune cells, sustaining cytokine-mediated tissue injury and contributing to late normal tissue toxicity [31]. Because RILA interrogates systemic lymphocyte apoptosis rather than tumor biology, it does not assess tumor radiosensitivity and does not predict tumor control. Its clinical relevance lies specifically in the prediction of patient-specific normal tissue toxicity. Multiple clinical cohorts, including prospective validation studies, have demonstrated that low RILA values are independently associated with an increased risk of clinically significant late toxicity, particularly fibrosis following breast and prostate radiotherapy. RILA’s principal limitations include lack of tissue specificity and inability to capture key radiosensitivity dimensions such as DNA repair kinetics or chromosomal misrepair. Nevertheless, RILA serves as a foundational benchmark for functional toxicity prediction and a reference platform for integration with complementary assays and germline genetic biomarkers in emerging hybrid models. In a large prospective study of 399 patients, radiation-induced apoptosis of CD4^+^ and particularly CD8^+^ T-lymphocytes was shown to significantly predict grade ≥2 late toxicity after radiotherapy, supporting its potential as a functional biomarker of individual radiosensitivity [12]. Similarly, in a prospective matched cohort study of 118 radiation-induced sarcoma (RIS) patients and 229 controls, lower CD8^+^ T-lymphocyte RILA was significantly associated with an increased risk of developing RIS [32]. For 49 patients with localized prostate cancer, in line with earlier RILA studies, pre-treatment CD8^+^ RILA significantly predicted late urinary toxicity, particularly patient-reported IPSS increase, while no significant association was observed with clinician-graded toxicity or survival outcomes [33]. Consistently, a large prospective multicenter trial in breast cancer further validated this approach, demonstrating that pre-treatment CD8^+^ radiation-induced lymphocyte apoptosis measured by flow cytometry independently predicted grade ≥2 late breast fibrosis after adjuvant radiotherapy, with a high negative predictive value for clinically significant toxicity [34]. In a long-term validation study of breast cancer patients, radiation-induced lymphocyte apoptosis was assessed by flow cytometric quantification of apoptosis in CD4^+^, CD8^+^, and NK lymphocyte subpopulations from peripheral blood following ex vivo irradiation with 8 Gy and 48 h incubation, demonstrating that low CD4^+^ RILA independently predicted late subcutaneous fibrosis and telangiectasia after radiotherapy [35]. Among currently available functional radiosensitivity assays, RILA remains one of the most clinically validated approaches, supported by multiple prospective studies, multicenter cohorts, and relatively reproducible associations with late normal tissue toxicity, particularly fibrosis after breast and prostate radiotherapy. Its comparatively strong negative predictive value and standardized flow-cytometric implementation have contributed to its emergence as a reference platform for functional toxicity prediction, although broader prospective validation and integration into clinical workflows are still needed.

### 2.2. G2 Chromosomal Radiosensitivity Assay

The G2 chromosomal radiosensitivity assay is a cytogenetic test designed to evaluate how effectively cells repair DNA double-strand breaks during the G2 phase of the cell cycle, a period in which post-replicative repair fidelity and checkpoint integrity are critical [13]. Peripheral blood lymphocytes or cultured fibroblasts are stimulated to proliferate and are synchronized to enrich for the G2 phase. Cells are subsequently irradiated and harvested for metaphase preparation. Chromatid-type aberrations, including chromatid breaks, gaps, and exchanges, are scored across a predefined number of metaphases, and a G2 score is calculated as the mean number of aberrations per cell. Elevated G2 scores reflect impaired resolution of radiation-induced DNA damage in late cell cycle and/or defective G2/M checkpoint control, allowing cells to progress into mitosis with unrepaired or misrepaired lesions that manifest as chromatid damage. Mechanistically, this phenotype implicates deficiencies in key DDR components, including ATM-mediated signaling, homologous recombination fidelity, and checkpoint enforcement [3]. Clinically, increased G2 radiosensitivity has been associated with a higher incidence of late normal tissue toxicity, particularly fibrosis and telangiectasia. More recent studies indicate that the predictive performance of the G2 assay can be enhanced through integration with germline genetic biomarkers and complementary functional readouts. Variants in DDR genes such as *ATM* and *BRCA1/2* have been shown to modulate chromosomal radiosensitivity, and combined functional–genetic models improve toxicity risk stratification compared with either approach alone [5]. Although the G2 assay does not directly predict tumor control or survival, its clinical relevance may indirectly extend to oncologic outcomes through its association with treatment-limiting toxicity. However, substantial barriers hinder the routine clinical implementation of the G2 assay. Its technical complexity, including prolonged cell culture, precise synchronization, labor-intensive metaphase preparation, and manual scoring, limits throughput and contributes to inter-operator and inter-laboratory variability [36]. Nevertheless, the G2 chromosomal radiosensitivity assay remains a biologically informative reference platform for assessing chromosomal misrepair and continues to play an important role in research efforts aimed at integrating functional and genetic predictors of normal tissue radiosensitivity. Early clinical studies have demonstrated that increased G2 chromosomal radiosensitivity in peripheral blood lymphocytes is associated with severe acute skin reactions following breast radiotherapy, supporting a link between impaired post-irradiation chromosomal repair and normal tissue radiosensitivity, although predictive power for late toxicity was limited [37]. Similarly, using a lymphocyte-based chromosomal radiosensitivity assay, Hoeller et al. demonstrated that breast cancer patients with elevated radiation-induced chromosomal aberrations exhibited a significantly higher annual risk of late fibrosis following radiotherapy [38]. In patients treated with pelvic radiotherapy for gynecologic malignancies, increased G2 chromosomal radiosensitivity in peripheral blood lymphocytes was significantly associated with a higher risk of late normal tissue toxicity, including gastrointestinal, genitourinary, and fibrotic complications [39]. More recently, a matched case–control study in breast cancer patients demonstrated that reduced G2 chromosomal radiosensitivity in peripheral blood lymphocytes was independently associated with late radiation-induced breast fibrosis, supporting the relevance of G2/M checkpoint dysfunction in normal tissue radiosensitivity [40]. In contrast, a prospective study using a modified G2 assay with caffeine-induced checkpoint abrogation found no association between pre-treatment individual radiosensitivity and acute radiation dermatitis, highlighting the limited predictive value of G2-based assays for acute skin toxicity [41]. Despite their strong mechanistic rationale, functional radiosensitivity assays face several important translational limitations that have restricted routine clinical implementation. Predictive performance varies substantially across studies due to differences in assay protocols, irradiation conditions, biological material, scoring methodologies, and clinical endpoints. Many assays remain labor-intensive, require specialized infrastructure, or exhibit significant inter-laboratory variability, limiting scalability and reproducibility. In addition, most studies are retrospective or are based on relatively small and heterogeneous patient cohorts, while prospective multicenter validation remains limited for many platforms. Another major challenge is that radiosensitivity represents a multifactorial and dynamic phenotype influenced not only by intrinsic DNA repair capacity, but also by systemic inflammation, treatment context, immune status, microenvironmental factors, and dosimetric parameters, which are not captured by single-assay approaches. Collectively, these limitations help explain why no individual functional assay has yet achieved widespread clinical adoption despite decades of investigation.

### 2.3. Micronucleus (MN) Assay

The cytokinesis-block micronucleus (CBMN) assay is a cytogenetic method that quantifies radiation-induced chromosomal damage by measuring the frequency of micronuclei in binucleated cells following irradiation [42]. Micronuclei arise from acentric chromosome fragments generated by misrepaired DNA double-strand breaks or from whole chromosomes that fail to segregate properly during mitosis. In standard protocols, peripheral blood lymphocytes are stimulated to proliferate, exposed to cytochalasin B to block cytokinesis and yield binucleated cells and subsequently scored for micronuclei per binucleated cell. Mechanistically, micronucleus formation reflects an integrated phenotype of genomic instability rather than a single repair defect. MN yield therefore captures the cumulative consequences of imperfect DSB repair, checkpoint dysfunction, replication stress, and mitotic errors. As such, the assay complements apoptosis-based platforms such as RILA and checkpoint-focused assays such as G2 by interrogating downstream chromosomal consequences of radiation exposure. Because the assay is typically performed on normal lymphocytes, it assesses patient radiosensitivity rather than tumor response. Using a lymphocyte-based micronucleus assay, De Ruyck et al. demonstrated that increased radiation-induced chromosomal damage was significantly associated with a higher risk of late normal tissue toxicity following pelvic radiotherapy for gynecologic cancers [39]. A major limitation of the MN assay has historically been variability arising from differences in culture conditions, staining protocols, and manual scoring. However, recent advances in standardization frameworks and automated or AI-assisted scoring have begun to improve reproducibility and inter-laboratory concordance. Wills et al. demonstrated that imaging flow cytometry combined with deep learning enables robust, inter-laboratory automation of the CBMN assay, achieving high concordance with expert manual scoring and substantially improving the throughput and reproducibility of micronucleus detection [43]. Despite these advances, individual-level predictive performance remains moderate across heterogeneous clinical cohorts, and prospective multicenter validation is still required.

### 2.4. Clonogenic Survival Assay

The clonogenic survival assay is considered the gold standard method for quantifying long-term reproductive cell survival following irradiation. In this assay, cells are exposed to graded radiation doses, plated at low density, and allowed to proliferate for one to three weeks. Colonies containing a predefined minimum number of cells—commonly ≥50—are subsequently counted, and survival curves are generated, often modeled using linear–quadratic fitting [44]. Mechanistically, clonogenic survival integrates the cumulative biological consequences of radiation exposure, including unrepaired or misrepaired DNA double-strand breaks, lethal chromosomal aberrations, mitotic catastrophe, checkpoint failure, and irreversible loss of proliferative capacity [45]. Because clonogenic death represents the final common pathway of multiple upstream defects, clonogenic radiosensitivity provides a comprehensive functional measure of intrinsic cellular radiation response. When performed on patient-derived fibroblasts or lymphocytes, the assay reflects normal tissue radiosensitivity rather than tumor control. Early foundational studies demonstrated that reduced clonogenic survival of patient derived fibroblasts—often quantified as survival fraction at 2 Gy (SF2)—can be associated with an increased risk of severe late normal tissue toxicity, including fibrosis and necrosis, although not all cohorts show strong correlations [46]. In breast cancer patients, Brock et al. reported that increased intrinsic radiosensitivity of normal skin fibroblasts, measured by clonogenic survival (SF2), is associated with a higher risk of late skin telangiectasia following radiotherapy, while acute reactions showed no consistent correlation [47]. Alsbeih et al. demonstrated that reduced clonogenic survival of patient-derived skin fibroblasts (lower SF2) was significantly associated with the development of severe late normal tissue complications following definitive radiotherapy across multiple tumor sites [48]. In contrast, Rudat et al. found that clonogenic radiosensitivity of patient-derived fibroblasts, measured as surviving fraction at 2 Gy, did not correlate with the severity of acute skin or mucosal toxicity in head-and-neck cancer patients undergoing radiotherapy [49]. Clonogenic assay is poorly suited for routine clinical application as long assay duration, low throughput, variable growth characteristics of primary patient-derived cultures, and sensitivity to technical conditions limit scalability and reproducibility. Consequently, clonogenic survival assays are primarily retained as mechanistic reference standards rather than deployable clinical biomarkers.

### 2.5. γ-H2AX Foci Kinetics

The γ-H2AX foci assay is a molecular approach for quantifying DSBs and monitoring repair kinetics following irradiation. DSB induction triggers rapid phosphorylation of the histone variant H2AX at serine 139 by ATM, ATR, and DNA-PKcs, generating γ-H2AX and producing discrete nuclear foci that can be visualized by immunofluorescence microscopy or quantified using high content imaging and flow-cytometric platforms. Mechanistically, γ-H2AX functions as a central chromatin scaffold for DNA damage response signaling, facilitating the recruitment and retention of repair factors at sites of damage. Clearance of γ-H2AX foci therefore requires not only successful DSB repair but also restoration of chromatin structure. Persistent γ-H2AX foci at later time points are interpreted as a marker of impaired repair capacity, defective signaling, or chromatin constraints that limit damage resolution. Clinically, delayed γ-H2AX foci clearance has been associated with increased susceptibility to late normal tissue toxicity across multiple disease sites, including breast, prostate, and head-and-neck radiotherapy [50,51,52]. Associations with survival outcomes are thought to be largely indirect, arising from increased toxicity, treatment interruptions, and compromised dose delivery. When applied to normal cells, γ-H2AX kinetics therefore function primarily as predictors of patient-specific toxicity risk and indirect treatment outcomes. Their application to tumor cells or tumor-derived organoids represents a distinct use case focused on tumor radiosensitivity and should be conceptually separated from normal tissue assays to avoid interpretive conflation [52]. Key translational challenges include pre-analytic variability, fixation timing, image acquisition and quantification pipelines, and biological confounders such as chromatin organization. Nevertheless, γ-H2AX foci kinetics remain some of the most mechanistically direct, scalable, and time-efficient functional assays for assessing DNA repair competence and represent a central component of emerging hybrid radiosensitivity platforms integrating functional, genetic, and tissue-specific information [53]. Bourton et al. have indicated that γ-H2AX foci persistence in normal peripheral blood lymphocytes functioned as a predictive biomarker of radiotherapy-induced normal tissue toxicity, with indirect implications for tumor control through treatment tolerance rather than intrinsic tumor radiosensitivity [54]. In a retrospective cohort of radiotherapy patients with extreme normal tissue toxicity, prolonged γ-H2AX signal retention and altered repair kinetics in ex vivo-irradiated peripheral blood lymphocytes (quantified by increased unrepairable damage fraction and reduced repair rate) accurately identified intrinsically radiosensitive individuals, supporting γ-H2AX-based functional assays as predictors of severe radiotherapy toxicity [55]. Leong et al., in a retrospective clinical cohort, measured γ-H2AX repair kinetics and γ-H2AX/53BP1 colocalization in ex vivo-irradiated peripheral blood lymphocytes and hair follicles and distinguished patients who later developed severe late radiotherapy toxicity from matched controls, supporting γ-H2AX-based functional assays as predictors of intrinsic radiosensitivity [56]. In a cohort of pelvic cancer patients treated with definitive radiotherapy, a higher γ-H2AX foci decay ratio (FDR ≥ 0.59) measured in ex vivo-irradiated peripheral blood lymphocytes was independently associated with an increased risk of late genitourinary and gastrointestinal toxicity, supporting its potential role as a predictive biomarker of normal tissue radiosensitivity [57]. In a prospective cohort of prostate cancer patients treated with external beam radiotherapy, γ-H2AX foci decay ratio measured in ex vivo-irradiated peripheral blood lymphocytes was independently associated with grade ≥2 late radiation toxicity, outperforming conventional dose–volume parameters as a predictor of normal tissue radiosensitivity [58]. Two years later the same group, in a prospective cohort of prostate and gynecological cancer patients treated with contemporary pelvic radiotherapy, measured the γ-H2AX foci decay ratio in ex vivo-irradiated peripheral blood lymphocytes and no correlation with physician-assessed or patient-reported late toxicity was found, indicating reduced predictive value of this assay in the setting of modern, highly conformal radiotherapy techniques [59]. In patient-derived lymphoblastoid cell lines from individuals with severe radiotherapy-induced toxicity, γ-H2AX foci kinetics were largely comparable to controls at the cohort level, but a rare radiosensitive case with profoundly delayed DNA double-strand break repair was identified, highlighting the assay’s utility for detecting extreme intrinsic radiosensitivity rather than population-wide risk [60]. Notably, Werbrouck et al. report a negative clinical result, demonstrating that γ-H2AX foci kinetics in peripheral blood T-lymphocytes did not predict late normal tissue toxicity in gynecological radiotherapy patients, highlighting disease- and context-specific limitations of the assay [61].

Unlike γ-H2AX assays performed in normal cells, where repair kinetics primarily reflect host DNA damage response capacity and toxicity risk, tumor γ-H2AX measurements are strongly influenced by microenvironmental factors such as hypoxia, proliferation, and intratumoral heterogeneity, complicating their direct interpretation as intrinsic radiosensitivity biomarkers. In patients with locally advanced rectal cancer undergoing preoperative concurrent chemoradiation, dynamic increases in γ-H2AX levels in peripheral blood mononuclear cells during treatment were found to be associated with radiologic tumor response and pathologic complete response, suggesting a potential role for γ-H2AX as a biomarker of treatment response rather than normal tissue radiosensitivity [62]. In a multicenter cohort of 311 resected pancreatic ductal adenocarcinomas, high tumoral γ-H2AX expression assessed by immunohistochemistry was independently associated with worse overall survival and enrichment of the basal-like subtype, supporting the idea of using γ-H2AX as a prognostic tumor biomarker rather than a functional radiosensitivity assay [63].

### 2.6. Comet Assay (Single-Cell Gel Electrophoresis)

The comet assay, also known as single-cell gel electrophoresis, is a sensitive method for detecting DNA strand breaks at the single-cell level following irradiation. Cells are embedded in agarose, lysed to remove membranes and proteins, and subjected to electrophoresis under alkaline or neutral conditions. Fragmented DNA migrates away from the nuclear core, forming a characteristic “comet tail”, the extent of which reflects the burden of DNA damage. Quantitative parameters such as tail length, tail moment, or percentage of DNA in the tail are commonly used to assess damage induction and repair kinetics [17,64]. Mechanistically, the comet assay captures both initial DNA damage and the efficiency of repair processes over time. Under alkaline conditions, the assay detects single-strand breaks, alkali-labile sites, and transient repair intermediates, whereas neutral comet variants preferentially reflect double-strand breaks. As such, comet-based radiosensitivity phenotyping integrates multiple aspects of DNA damage processing rather than isolating a single repair pathway. When performed on patient-derived lymphocytes or fibroblasts, the assay reflects normal tissue radiosensitivity rather than tumor response [65]. Clinical studies evaluating the predictive value of comet assays have yielded mixed results. Some analyses have reported associations between residual DNA damage after in vitro irradiation and severe late normal tissue reactions, suggesting a potential role as a predictor of normal tissue radiosensitivity, whereas others have not found the assay to be a useful predictor of clinical toxicity. Using the alkaline comet assay, Alapetite et al. demonstrated that peripheral blood lymphocytes from cancer patients who developed severe radiotherapy toxicity exhibited delayed repair of radiation-induced DNA strand breaks compared with normal reactors, indicating impaired DNA repair capacity in radiosensitive individuals [66]. Müller et al. observed that patients experiencing severe radiotherapy-induced toxicity displayed higher levels of residual DNA damage in lymphocytes after irradiation, suggesting reduced DNA repair capacity in radiosensitive individuals [67]. In a recent study, Ocolotobiche et al. demonstrated that breast cancer patients who developed acute radiotherapy-induced skin toxicity showed significantly reduced DNA repair capacity and increased residual DNA damage in peripheral blood lymphocytes measured by the alkaline comet assay, supporting the idea of impaired DNA repair being a contributor to individual radiosensitivity [68]. Popanda et al. have reported that, although the alkaline comet assay identified interindividual differences in radiation-induced DNA damage and repair capacity in breast cancer patients, these parameters showed only limited correlation with acute skin toxicity during radiotherapy [69]. On the same side, Adamczyk et al. evaluated DNA damage and repair kinetics in peripheral blood lymphocytes of cervical and laryngeal cancer patients using the alkaline comet assay and found that comet parameters showed only limited and inconsistent associations with acute and late radiotherapy-induced normal tissue toxicity, suggesting the limited predictive value of the assay for clinical radiosensitivity [70]. Overall, impaired DNA damage repair kinetics measured by comet assays may be linked to increased treatment-related toxicity in selected settings, though the evidence remains heterogeneous. Despite its sensitivity and relative technical simplicity, the comet assay faces limitations that hinder routine clinical implementation. Variability in assay conditions, including electrophoresis parameters, scoring metrics, and operator-dependent image analysis, contributes to inconsistent performance across laboratories [71]. Compared with more clinically mature assays such as RILA, the comet assay currently remains primarily a mechanistic and exploratory research tool. Although it provides sensitive assessment of DNA damage and repair kinetics, clinical studies have produced heterogeneous and frequently inconsistent results, and reproducibility across laboratories remains limited due to methodological variability. Consequently, its current translational utility for individualized radiotherapy decision-making remains uncertain.

### 2.7. Patient-Derived Organoid Models in Radiotherapy Response

Patient-derived organoid systems represent an emerging class of functional radiobiology platforms capable of modeling tissue-specific radiation response using three-dimensional epithelial structures generated from adult stem cells. Unlike conventional two-dimensional culture systems, organoids preserve important aspects of tissue architecture, cellular heterogeneity, and lineage organization, thereby providing biologically relevant models for studying both tumor radiosensitivity and normal tissue radiation injury. Current evidence is strongest for tumor response prediction and personalized treatment modeling, while applications in normal tissue toxicity prediction remain comparatively early and less clinically validated. In radiotherapy research, this is relevant both for modeling tumor treatment response and for investigating mechanisms of radiation-induced normal tissue injury, particularly epithelial stem-cell depletion and impaired tissue regeneration [72]. Organoids are typically established from patient-derived biopsies or surgical specimens, embedded in extracellular matrix, and expanded under defined growth-factor conditions. Following irradiation, radiosensitivity can be quantified using endpoints such as organoid-forming efficiency, viability, growth kinetics, morphology, and long-term regenerative potential. Importantly, organoid systems can also be combined with molecular DNA damage response readouts, including γ-H2AX foci analysis, thereby linking epithelial survival and regenerative capacity to underlying repair competence. This distinguishes organoid assays from reductionist single-endpoint platforms by enabling simultaneous assessment of stem-cell survival, tissue repair, and mechanistic radiation response [73]. Preclinical studies provide strong biological support for this approach. Intestinal organoid work has shown that organoid dose–survival curves reflect the inherent radiosensitivity of the tissue of origin and the stem-cell compartment, supporting the use of organoids as quantitative models of organ-level radiation sensitivity [18]. In a translational study, organoids derived from multiple cancer types were used to assess sensitivity to chemotherapy and radiation and, in a prospective clinical case, organoid response accurately predicted treatment outcome in a patient with metastatic colorectal cancer, supporting their role as functional predictive platforms for radiotherapy response [74]. Specifically, in locally advanced rectal cancer, a prospective co-clinical study demonstrated that patient-derived organoids established prior to treatment accurately predicted response to neoadjuvant chemoradiotherapy, with reported accuracy exceeding 80% [75]. In parallel, patient-derived pancreatic cancer organoids have demonstrated clear radiation dose–response relationships and inter-individual heterogeneity, supporting their potential as functional platforms for modeling personalized radiotherapy response [76]. However, direct clinical validation for toxicity prediction remains limited. Their principal strengths are tissue specificity, the ability to interrogate epithelial stem-cell regeneration, and compatibility with integrated functional and molecular readouts. Their current limitations include variability in tissue acquisition and culture success, lack of stromal, immune, and vascular compartments in many systems, absence of standardized irradiation and scoring pipelines, and the scarcity of prospective studies correlating organoid radiosensitivity with patient-reported or physician-graded toxicity outcomes after radiotherapy. Although organoid platforms have shown considerable promise for predicting tumor response to radiotherapy and chemoradiotherapy, direct clinical validation for normal tissue toxicity prediction remains limited. Most currently available studies involve tumor-derived organoids and focus primarily on treatment response rather than prospective correlation with patient-specific toxicity outcomes. Consequently, while normal tissue organoid systems represent an important future direction, their translational role in individualized toxicity prediction remains largely investigational at present. As co-clinical studies mature and standardized workflows become available, normal tissue organoids may become an important component of personalized radiotherapy, particularly when integrated with genetic or molecular biomarkers.

### 2.8. Genetic Biomarkers and Hybrid Functional–Genomic Models

While functional radiosensitivity assays provide direct phenotypic measurements of cellular responses to ionizing radiation, germline genetic biomarkers offer complementary insight into inherited determinants of individual radiosensitivity. Variants in genes involved in the DNA damage response, cell-cycle regulation, and tissue remodeling pathways have been associated with differential susceptibility to radiation-induced normal tissue toxicity [8,77]. Early radiogenomic studies identified SNPs in key genes such as *ATM*, *TGFB1*, *BRCA1*, and *BRCA2* as potential contributors to variability in late radiation effects [10,78]. However, the predictive performance of individual genetic variants has generally been modest across heterogeneous patient populations, reflecting the complex and polygenic nature of radiosensitivity. As a result, increasing attention has focused on integrative approaches that combine germline genetic information with functional assays that capture the downstream biological consequences of these variants [29,79]. Such hybrid functional–genomic models aim to link inherited susceptibility with measurable cellular phenotypes, thereby providing a more comprehensive framework for predicting normal tissue toxicity risk and advancing personalized radiotherapy strategies. In nasopharyngeal carcinoma, Alsbeih et al. performed genotyping of 45 candidate SNPs in DNA damage response and repair genes, including *ATM* (rs1801516), *TGFB1* (C-509T, T869C), *XRCC1*, and *XRCC3*, using PCR-based sequencing of peripheral blood DNA [80]. Patients carrying “risk alleles” exhibited significantly higher rates of severe late fibrosis and radiation-induced morbidity when compared with non-carriers. Multiple variants, including *ATM* rs1801516 and *TGFB1* C-509T, contributed cumulatively to this toxicity risk, highlighting the polygenic nature of radiosensitivity. In breast cancer, Seibold et al. analyzed SNPs in *XRCC1* codon 399 and *XRCC3* codon 241 using PCR-RFLP and Sanger sequencing. Patients with these variant alleles had an increased incidence of late skin toxicity, such as fibrosis and telangiectasia, following radiotherapy [81]. In a large prospective genome-wide association study of 1640 breast cancer patients from the REQUITE cohort, Jandu et al. tested over seven million SNPs for association with chronic radiation-related toxicity measured two years after whole-breast radiotherapy, identifying eight variants reaching genome-wide significance for specific endpoints such as nipple retraction, breast oedema, tissue induration, and arm lymphoedema; estimated heritability for these traits ranged from approximately 25% to 39%, providing robust evidence that common germline variants contribute to long-term normal tissue toxicity [78]. In a cohort of head-and-neck squamous cell carcinoma patients treated with radiotherapy, targeted next-generation sequencing of matched normal tissue identified germline variants in genes including *TSC2*, *HLA-A*, *TET2*, *GEN1*, and *NCOR2* that were significantly associated with long-term normal tissue toxicity (e.g., dysphagia, xerostomia, fibrosis). Patients with increased late toxicity clustered by CTCAE v5.0 criteria also showed distinct clinical outcomes, with some variants (e.g., *TSC2*, *FANCD2*, *PPP1R15A*) correlated with improved overall survival and progression-related outcomes and others (e.g., *HLA-DMA/HLA-DMB* group) linked to higher locoregional recurrence risk, suggesting potential utility of these germline biomarkers in stratifying toxicity and therapeutic outcomes after radiotherapy. Hybrid functional–genomic approaches have also been explored [82]. In prostate cancer, Mališić et al. combined SNP genotyping of *TGFB1* and *XRCC* family variants with RILA assays where lymphocytes were irradiated ex vivo and apoptosis was quantified via flow cytometry [79]. Integration of SNP risk alleles with RILA measurements improved prediction of acute genitourinary and gastrointestinal toxicity; patients with both low RILA values and risk alleles experienced the highest toxicity. This approach illustrates how combining germline susceptibility with phenotypic functional assays can enhance risk stratification. Large-scale genome-wide analyses have further identified novel susceptibility loci. In head-and-neck cancer, Schack et al. conducted a genome-wide associated study (GWAS) using high-density SNP arrays to associate genetic variants with prospectively collected toxicity endpoints. They identified multiple loci, specific locations on chromosomes containing genes or regulatory elements, that were significantly associated with late fibrosis, mucositis, and xerostomia. While the effect sizes of individual loci were modest, combining information across loci into polygenic risk scores improved predictive performance. These loci act as genomic “addresses” where inherited variation can influence radiation-induced tissue responses [83]. Together, these studies demonstrate that germline genetic biomarkers, whether assessed individually, in combination, or integrated with functional assays, can provide insight into patient-specific radiosensitivity. Single SNPs typically have modest predictive value, but polygenic and hybrid functional–genomic approaches show promise for more comprehensive prediction of normal tissue toxicity, which could ultimately guide personalized radiotherapy strategies. In an international multi-center prostate cancer cohort from the prospective REQUITE study, researchers developed an interaction-aware polygenic risk score (PRSi) by analyzing SNP–SNP combinations from 43 literature-identified genetic variants and deriving risk and protection scores for late toxicity endpoints (rectal bleeding, urinary frequency, hematuria, nocturia, and decreased urinary stream). The resulting PRSi distributions differed significantly between patients with and without toxicity and demonstrated better discrimination than traditional summed polygenic risk scores, with area under the receiver operating characteristic (ROC) curves ranging from approximately 0.61 to 0.78 across toxicity outcomes, supporting the incorporation of SNP–SNP interaction information into genetic risk models for radiotherapy toxicity prediction [84].

Collectively, the currently available functional assays differ substantially in mechanistic specificity, technical complexity, scalability, and degree of clinical validation. RILA presently has the strongest clinical evidence base, particularly for the prediction of late fibrosis, supported by prospective multicenter studies and relatively consistent reproducibility across cohorts. γ-H2AX-based approaches and G2 chromosomal assays possess strong mechanistic rationale and have shown predictive associations in selected settings, but results remain more heterogeneous across disease sites and treatment modalities. In contrast, assays such as comet analysis and clonogenic survival testing remain primarily mechanistic or research-oriented due to technical complexity, limited standardization, and inconsistent predictive performance. Emerging organoid platforms offer important opportunities for tissue-specific modeling but currently lack sufficient prospective clinical validation for routine implementation. These differences highlight the need to distinguish mechanistic relevance from translational readiness when evaluating radiosensitivity biomarkers.

**Table 1:** Functional assays for normal tissue radiosensitivity: Methodology and associations with toxicity.

Assay	Method	Results on the Patient	Cancer Type(s)	Reference
Radiation-induced lymphocyte apoptosis (RILA)	Flow cytometry measuring CD4^+^ and CD8^+^ T-lymphocyte apoptosis after 8 Gy ex vivo irradiation of peripheral blood.	Low RILA identifies patients at higher risk of developing significant late radiation toxicity.	Breast, head-and-neck, genitourinary, gastrointestinal, lymphomas (Hodgkin and non-Hodgkin), gynecologic cancers, lung cancer, skin, soft tissue.	[12]
	Flow cytometry measuring CD8^+^ T-lymphocyte apoptosis after 8 Gy ex vivo irradiation of peripheral blood.	Lower RILA levels were significantly associated with an increased risk of radiation-induced sarcoma.	Breast, head-and-neck, Pelvic tumors, other locations.	[32]
	Flow cytometry measuring CD8^+^ T-lymphocyte apoptosis after 8 Gy ex vivo irradiation of peripheral blood.	Lower pre-treatment CD8^+^ RILA significantly predicted greater late urinary toxicity, particularly increased IPSS (patient-reported) scores, while RILA was not predictive of graded GI toxicity or survival outcomes.	Prostate.	[33]
	Flow cytometry-based quantification of CD8^+^ T-lymphocyte apoptosis after ex vivo irradiation (8 Gy) of peripheral blood collected before radiotherapy.	Low RILA values were independently associated with a higher risk of grade ≥2 late breast fibrosis after adjuvant radiotherapy. High RILA showed a strong negative predictive value (≈91%) for clinically significant fibrosis.	Breast.	[34]
	Flow cytometry-based quantification of radiation-induced apoptosis in CD4^+^, CD8^+^, and CD3^−^/CD8^+^ (NK) lymphocyte subpopulations isolated from peripheral blood (ex vivo irradiated with 8 Gy).	Low CD4^+^ RILA was independently associated with increased risk of late subcutaneous fibrosis and telangiectasia after breast radiotherapy at >10-year follow-up. Patients in the lowest CD4^+^ RILA tertile showed OR 3.48 for fibrosis and OR 8.60 for telangiectasia, with high negative predictive value for severe late toxicity. CD8^+^ and NK cell RILA were not significantly associated with late toxicity.	Breast.	[35]
G2- Chromosomal radiosensitivity assay	Cytogenetic analysis of chromatid breaks in G2 phase lymphocytes after irradiation.	Higher G2 radiosensitivity was associated with severe acute skin toxicity after breast radiotherapy; limited and non-significant association with late fibrosis or telangiectasia.	Breast.	[37]
	Peripheral blood lymphocytes irradiated in vitro (6 Gy), stimulated to first metaphase, and lethal chromosomal aberrations (acentric fragments and dicentrics) scored by cytogenetic analysis.	High chromosomal radiosensitivity was associated with a 2.3-fold higher annual risk of late breast fibrosis	Breast.	[38]
	Chromatid breaks per cell scored in peripheral blood lymphocytes stimulated and irradiated in vitro in G2 phase (0.4 Gy).	Higher G2 chromosomal radiosensitivity was significantly associated with late normal tissue toxicity after pelvic radiotherapy, with patients in the high G2 group showing a 9.2-fold increased annual risk of complications.	Gynecologic.	[39]
	Chromatid breaks and gaps scored by cytogenetic analysis and expressed as a G2 radiosensitivity index in peripheral blood lymphocytes irradiated in vitro in G2 phase (1 Gy) with and without caffeine.	Patients who developed grade ≥2 late breast fibrosis showed significantly lower G2 assay values, indicating impaired G2/M checkpoint function, the G2 assay remained independently associated with fibrosis after adjustment for clinical covariates.	Breast.	[40]
	Peripheral blood lymphocytes irradiated in vitro (1 Gy); G2 chromosomal aberrations quantified with and without caffeine-induced G2/M checkpoint abrogation and expressed as an individual radiosensitivity (IRS) ratio.	Pre-treatment IRS did not predict acute radiation dermatitis during whole-breast radiotherapy.	Breast.	[41]
Micronucleus assay	Peripheral blood lymphocytes irradiated in vitro, cultured with cytokinesis block, and binucleated cells scored for micronuclei.	Higher radiation-induced micronucleus frequencies were significantly associated with increased late normal tissue toxicity (CTC ≥ 2), including gastrointestinal, genitourinary, vaginal, and fibrotic complications after pelvic radiotherapy.	Gynecologic.	[39]
Colony-Forming Survival Assay	Early-passage skin fibroblasts obtained from patient biopsies irradiated in vitro and surviving fraction at 2 Gy (SF2) derived from clonogenic survival curves fitted using a linear–quadratic model.	Lower fibroblast SF2 values (higher radiosensitivity) were associated with increased late skin toxicity, particularly telangiectasia, whereas no consistent association was observed with acute skin reactions.	Breast.	[47]
	Primary skin fibroblasts obtained from patient punch biopsies irradiated in vitro and surviving fraction at 2 Gy (SF2) derived from clonogenic survival curves using linear–quadratic fitting.	Patients who developed moderate to severe late normal tissue complications showed significantly lower fibroblast SF2 values compared with patients without late toxicity.	Breast, prostate, head-and-neck, cervix, bladder, anal canal, thyroid.	[48]
	Primary human fibroblasts derived from skin or periodontal biopsies irradiated in vitro and surviving fraction at 2 Gy (SF2) calculated from clonogenic survival curves fitted with the linear–quadratic model.	Despite marked inter-individual variation in fibroblast radiosensitivity, no correlation was observed between SF2 and acute normal tissue toxicity (skin or mucosa) in patients with head-and-neck cancer treated with definitive radiotherapy.	Head-and-neck.	[49]
γ-H2AX/53BP1 foci kinetics	Quantification of γ-H2AX foci induction and residual foci persistence (24 h) following ex vivo 2 Gy γ-irradiation of peripheral blood lymphocytes using immunofluorescence microscopy.	Prolonged persistence of γ-H2AX foci strongly correlated with excessive acute and late normal tissue toxicity following radiotherapy. Impact on tumor control is indirect, mediated through increased toxicity, treatment interruptions, and dose-limiting effects rather than intrinsic tumor response.	Breast, head-and-neck, gynecologic, prostate.	[54]
	Quantification of γ-H2AX foci induction and repair kinetics in peripheral blood lymphocytes isolated from radiotherapy patients and controls following ex vivo γ-irradiation (2 Gy).	Patients with severe radiotherapy-induced normal tissue toxicity exhibited slower γ-H2AX decay, a higher fraction of unrepaired DNA damage, and impaired DSB repair capacity compared with normal reactors and controls. γ-H2AX kinetic parameters reliably identified an intrinsically radiosensitive phenotype. Tumor control outcomes were not assessed.	Mixed cancer cohort.	[55]
	Ex vivo irradiation of peripheral blood lymphocytes and eyebrow hair follicles from radiotherapy patients to assess the γ-H2AX foci peak response, repair rate, fraction of unrepaired damage, and γ-H2AX/53BP1 colocalization efficiency as indicators of DSB repair capacity.	In a retrospective cohort (16 radiosensitive vs. 12 matched controls), patients who developed severe late radiation toxicity exhibited defects in γ-H2AX repair kinetics and/or colocalization parameters compared with controls. The assay identified an intrinsically radiosensitive phenotype. Tumor control outcomes were not assessed.	Mixed cancer cohort.	[56]
	Peripheral blood collected prior to treatment ex vivo irradiated with 1 Gy X-ray and γ-H2AX foci quantification at 30 min and 4 h post-irradiation. FDR calculated as foci(30 min)/foci(4 h).	Foci decay ratio (FDR) ≥ 0.59 was independently associated with increased risk of late GU/GI toxicity (Grade ≥ 1). Tumor control was not assessed.	Cervical, vaginal, and anal.	[57]
	Peripheral blood lymphocytes were isolated from radiotherapy patients, irradiated ex vivo, and γ-H2AX foci were quantified at ~30 min and 24 h and the foci decay ratio (γ-FDR) was calculated as foci(30 min)/foci(24 h).	γ-H2AX < 3.41 (indicative of less efficient DNA double-strand break repair) was independently associated with significantly increased grade ≥2 late radiation toxicity suggesting impaired DNA repair proficiency as a critical predictor of late normal tissue toxicity. Tumor control outcomes were implied indirectly through prediction of toxicity risk affecting tolerance to curative radiotherapy, not derived directly from tumor response data in this study	Prostate.	[58]
	Ex vivo irradiation of isolated lymphocytes (1 Gy) and γ-H2AX foci quantified at 30 min and 24 h post-irradiation using immunofluorescence and automated deep learning-assisted image analysis; γ-FDR calculated as foci (30 min)/foci(24 h).	γ-FDR was not significantly associated with physician-assessed (CTCAE ≥ 2) or patient-reported moderate/severe late toxicity, nor with post-treatment global quality of life. The previously established threshold (γ-FDR < 3.41) did not predict toxicity. Tumor control outcomes were not assessed.	Prostate and gynecologic.	[59]
	Immunocytochemical quantification of γ-H2AX foci induction and repair kinetics in lymphoblastoid cell lines derived from radiotherapy patients with severe acute or late toxicity (RTOG ≥ 3) and matched controls following ex vivo γ-irradiation (2 Gy).	No significant difference in γ-H2AX repair kinetics was observed between radiosensitive patients and controls at the cohort level. However, one radiosensitive patient-derived cell line displayed markedly delayed γ-H2AX clearance, elevated basal foci levels, and defective DSB repair confirmed by PFGE, indicating the presence of a rare intrinsic DNA repair defect. Tumor control outcomes were not assessed.	Mixed cancer cohort.	[60]
	Quantification of γ-H2AX foci induction and repair kinetics in peripheral blood T-lymphocytes isolated from radiotherapy patients. Cells were irradiated ex vivo and residual foci and repair kinetics assessed by immunofluorescence microscopy.	No association was observed between γ-H2AX repair kinetics or residual foci levels and late normal tissue toxicity (CTCAE v3.0). T lymphocytes were not predictive of late radiotoxicity in this cohort. Tumor control was not assessed.	Gynecological.	[61]
	Longitudinal measurement of γ-H2AX activation in peripheral blood mononuclear cells collected at five time points (baseline, 24 h after first fraction, mid-treatment, end of treatment, and 6 weeks post-CCRT). γ-H2AX quantified by flow cytometry; activation ratios calculated relative to baseline.	Higher and dynamically increasing γ-H2AX activation during chemoradiation was observed in patients classified as responders by MRI tumor regression grade (mrTRG) and in those achieving pathologic complete response. Associations with long-term survival or local tumor control were not demonstrated. Toxicity correlations were not observed.	Rectal.	[62]
	Immunohistochemical assessment of γ-H2AX in resected tumor tissue (tissue microarrays); H score calculated based on staining intensity and percentage of positive tumor cells.	High tumoral γ-H2AX expression was independently associated with worse overall survival and increased frequency of the basal-like subtype. No radiotherapy-specific response or tumor control endpoint was evaluated.	Pancreatic ductal adenocarcinoma.	[63]
Neutral comet assay	Peripheral blood lymphocytes irradiated ex vivo (5 Gy) and DNA strand-break repair kinetics quantified using the alkaline comet assay by measuring residual DNA damage at multiple time points after irradiation.	Patients who developed severe radiotherapy toxicity exhibited delayed repair of radiation-induced DNA strand breaks and increased residual DNA damage compared with patients with normal reactions.	Breast; Hodgkin’s disease.	[66]
	Peripheral blood lymphocytes irradiated in vitro (0.25–2 Gy X-rays) and DNA damage and repair kinetics measured using the comet assay, with residual DNA damage after 3 h of repair used as a marker of DNA repair capacity.	Patients who developed severe normal tissue side-effects after radiotherapy showed significantly higher residual DNA damage after repair, indicating impaired DNA repair capacity and increased radiosensitivity.	Multiple tumor types (head-and-neck, lung, breast, cervix, prostate, esophagus).	[67]
	Peripheral blood lymphocytes from healthy volunteers and breast cancer patients were irradiated in vitro (6 Gy) and DNA damage and repair kinetics were quantified using the alkaline comet assay, with the damage index and residual DNA damage measured at 0, 60, and 120 min post-irradiation.	Breast cancer patients who developed acute radiotherapy-induced skin toxicity exhibited reduced DNA repair capacity and significantly higher residual DNA damage compared with patients without radiotoxicity.	Breast.	[68]
	Peripheral blood lymphocytes from breast cancer patients irradiated ex vivo with 5 Gy γ-rays, and DNA damage and repair kinetics quantified using the alkaline comet assay (tail moment) at 0, 15, and 30 min post-irradiation.	Commet assay parameters showed only limited correlation with acute radiation-induced skin toxicity, suggesting restricted predictive value for acute radiosensitivity.	Breast.	[69]
	Alkaline comet assay performed in peripheral blood lymphocytes irradiated ex vivo with 6 Gy X-rays, measuring endogenous, initial, and residual DNA damage (tail moment) after repair.	Comet assay parameters showed inconsistent and weak correlations with radiation-induced toxicity and were not reliable predictors of normal tissue reactions.	Cervical and laryngeal.	[70]
Patient-derived organoid response models	Ex vivo irradiation and chemoradiation of organoids derived from patient tumor samples, with assessment of growth and optical metabolic imaging.	Organoids recapitulated inter-patient variability in radiation response and were able to prospectively predict treatment outcome in a patient with metastatic colorectal cancer, including identification of resistance to single-agent therapy and response to combination treatment.	Colorectal (and other gastrointestinal).	[74]
	Ex vivo chemoradiotherapy testing of organoids derived from treatment-naïve rectal cancer patients enrolled in a clinical trial.	Organoid response strongly correlated with clinical response to chemoradiotherapy, achieving ~84% predictive accuracy, with high sensitivity and specificity.	Locally advanced rectal cancer.	[75]
	Ex vivo irradiation of patient-derived pancreatic cancer organoids (0–40 Gy) with viability assessment (ATP-based) and dose–response modeling over time.	Organoids demonstrated clear radiation dose–response relationships and inter-patient variability in radiosensitivity, supporting their use as functional models of individualized radiation response.	Pancreatic.	[76]
Genetic biomarkers and hybrid functional–genomic models	Genotyping of candidate SNPs in ATM (rs1801516), TGFB1 (C-509T, T869C), XRCC1, and XRCC3 using PCR-based assays from peripheral blood DNA.	Patients carrying “risk alleles” had significantly higher rates of severe late fibrosis and radiation-induced morbidity compared with non-carriers.	Nasopharyngeal carcinoma.	[80]
	SNP analysis of XRCC1 codon 399, XRCC3 codon 241 via PCR-RFLP and Sanger sequencing.	Patients with variant alleles had increased incidence of late skin toxicity (fibrosis, telangiectasia) following breast radiotherapy.	Breast.	[81]
	GWAS performed on 7,097,340 variants in 1640 patients with comprehensive clinical, treatment, and toxicity data, associations with chronic toxicity endpoints tested after adjustment for clinical/treatment covariates and population structure.	Identified eight SNPs significantly associated with long-term radiotherapy toxicity, including breast oedema, induration, nipple retraction, and arm lymphoedema	Breast.	[78]
	Targeted genotyping of candidate *SNPs* in *TSC2*, *HLA-A*, *TET2*, *GEN1*, and *NCOR2* from peripheral blood DNA using PCR-based assays.	Patients carrying risk variants in these genes had significantly higher rates of long-term normal tissue toxicities (fibrosis, mucositis, xerostomia)	Head-and-neck squamous cell carcinoma (HNSCC).	[82]
	Combination of SNP genotyping (*TGFB1*, *XRCC1/2/3*) and RILA, lymphocytes irradiated ex vivo, apoptosis measured via flow cytometry.	Integration of SNP risk alleles and RILA improved prediction of acute genitourinary and gastrointestinal toxicity, low RILA and risk alleles correlated with highest toxicity risk	Prostate.	[79]
	Genome-wide SNP genotyping using high-density arrays, association with prospectively collected toxicity endpoints.	Identified multiple loci (specific locations on chromosomes containing genes or genetic markers) significantly associated with late fibrosis, mucositis, and xerostomia	Head-and-neck.	[83]
	Genome-wide genotyping of literature-identified SNPs (from a panel of 43) followed by derivation of SNP-allele sets and calculation of interaction-aware polygenic risk scores (PRSi) from SNP–SNP combinations (risk and protection scores via logistic regression).	The interaction-aware PRSi distributions differed significantly between patients with and without late radiotherapy toxicity (e.g., rectal bleeding, urinary frequency, hematuria, nocturia, and decreased urinary stream), with PRSi outperforming classical summed PRS in discriminating toxicity (AUCs 0.61–0.78 across endpoints).	Prostate.	[84]

Despite strong mechanistic rationale, functional assays show variable predictive performance across clinical cohorts, reflecting technical variability and the inherently multi-factorial nature of radiosensitivity. No single assay consistently predicts clinical outcome, supporting the need for integrative approaches that combine multiple biological dimensions.

## 3. Tumor Radiosensitivity and Molecular Predictors of Treatment Response

While the previous sections focused primarily on normal tissue radiosensitivity, this section shifts attention to tumor-specific responses to radiation and the biomarkers used to predict treatment efficacy. The major tumor-specific biomarkers and genomic predictors associated with radiotherapy response are summarized in Table 2.

### 3.1. Tumor Clonogenic Radiosensitivity

The intrinsic capacity of a tumor to respond to ionizing radiation is fundamentally determined by the radiosensitivity of its clonogenic cell population, the subset of cells capable of sustaining long-term proliferation and tumor regrowth after treatment. In contrast to normal tissue assays that evaluate host sensitivity, tumor clonogenic radiosensitivity reflects the collective outcome of multiple tumor-intrinsic factors, including genomic instability, DNA repair proficiency, replication stress tolerance, and the microenvironmental context in which tumor cells reside. At the cellular level, clonogenic radiosensitivity embodies the balance between lethal DNA double-strand-break accumulation and the efficiency of DNA repair pathways such as non-homologous end-joining (NHEJ) and homologous recombination (HR). Tumors harboring deficiencies in HR or checkpoint control often demonstrate steep survival curve slopes, corresponding to high α and reduced β components within the linear–quadratic model, and are therefore more readily sterilized at clinically achievable doses. The integration of clonogenic principles into tumor control probability (TCP) modeling has formed the quantitative basis for modern radiotherapy treatment planning. TCP frameworks assume a Poisson probability of sterilizing all clonogens within the irradiated volume, mathematically linked to the linear–quadratic description of cell killing [85,86]. These models provide a mechanistic bridge between cellular-scale radiosensitivity and patient-scale tumor eradication, informing biologically optimized fractionation schedules and dose escalation in radioresistant disease. Clinically, clonogenic radiosensitivity has been correlated with radiotherapy outcome, as in a prospective study of cervical carcinoma, where ex vivo tumor SF2 values predicted local control and with highly radiosensitive tumors (low SF2) achieving markedly greater complete response rates following definitive irradiation [87]. In parallel, Björk-Eriksson et al. provided clinical evidence that intrinsic tumor clonogenic radiosensitivity predicts outcome: head-and-neck cancers exhibiting low SF_2_ in vitro achieved significantly higher complete response and locoregional control rates following curative radiotherapy, supporting the biological validity of clonogenic radiosensitivity as a determinant of treatment success [88]. In contrast to the positive findings, a subsequent clinical study using the modified Courtenay–Mills soft-agar clonogenic assay in head-and-neck squamous cell carcinoma demonstrated wide inter-tumor variability in SF_2_ but no significant association between tumor clonogenic radiosensitivity and locoregional control after curative radiotherapy [89]. In general, although clonogenic SF_2_ assays provided early evidence that intrinsic radiosensitivity may correlate with tumor control in certain clinical settings, their limited reproducibility, technical complexity, and inconsistent predictive value across studies have prevented their translation into routine clinical practice.

### 3.2. Tumor Hypoxia and Hypoxia Gene Signatures

Tumor hypoxia is a well-established determinant of radioresistance and an important source of inter-tumoral heterogeneity in radiotherapy response. Reduced oxygen availability limits the fixation of radiation-induced DNA damage, thereby decreasing the effectiveness of ionizing radiation and promoting tumor cell survival [90,91]. In addition to its direct radiobiological effects, hypoxia induces widespread transcriptional reprogramming mediated primarily by hypoxia-inducible factors (HIFs), leading to alterations in angiogenesis, metabolism, DNA repair, and cell survival pathways [92]. To capture the complexity and spatial heterogeneity of hypoxia within tumors, gene expression-based hypoxia signatures have been developed as surrogate biomarkers. These signatures integrate the expression of hypoxia-responsive genes and have demonstrated prognostic and predictive value across multiple cancer types [93]. A hypoxia metagene derived from head-and-neck cancer has shown that tumors with elevated hypoxia-associated transcriptional profiles exhibit increased radioresistance and inferior clinical outcomes, reinforcing the role of gene expression-based hypoxia signatures as predictive biomarkers for radiotherapy response and potential guides for hypoxia-targeted treatment strategies [94]. A 15-gene hypoxia classifier has been clinically validated by Toustrup et al. in head-and-neck cancer, where hypoxic tumors were associated with poorer radiotherapy outcomes, while patients derived benefit from hypoxia-modifying treatment [95]. Two years later, a clinically evaluated 26-gene hypoxia signature demonstrated that hypoxic tumors exhibit differential response to radiotherapy, with significantly improved tumor control following hypoxia-modifying treatment in laryngeal cancer, highlighting both its predictive value and tumor-type specificity [96]. It should be highlighted that, despite their promise, challenges remain regarding standardization, tumor specificity, and integration into clinical workflows.

### 3.3. Gene Expression Predictors

Gene expression-based models have emerged as a powerful approach to quantify intrinsic tumor radiosensitivity by capturing the coordinated activity of multiple biological pathways involved in radiation response. Among these, the Radiosensitivity Index (RSI), represents one of the most extensively studied and clinically translated genomic predictors of tumor radiosensitivity. RSI is a multigene expression model derived from the integration of gene expression profiles and clonogenic survival data (SF2) across cancer cell lines, incorporating genes involved in DNA damage response, cell-cycle regulation, apoptosis, and signal transduction. The RSI score is calculated using a rank-based algorithm based on the expression of ten genes, generating a continuous variable that reflects intrinsic cellular radiosensitivity. Lower RSI values correspond to a radiosensitive phenotype, whereas higher values indicate relative radioresistance. Initial development studies have demonstrated that RSI correlates with clonogenic survival following irradiation, establishing a biological link between gene expression patterns and radiation response [97,98]. Subsequent clinical validation studies have demonstrated that RSI is associated with radiotherapy outcomes across multiple tumor types. In breast cancer cohorts treated with radiotherapy, RSI-defined radiosensitive tumors showed significantly improved relapse-free survival compared with radioresistant tumors, while no association was observed in patients not receiving radiotherapy, supporting its role as a treatment-specific predictive biomarker [99]. Similarly, earlier multi-cohort validation studies in rectal, esophageal, and head-and-neck cancers showed that lower RSI values were associated with improved response to chemoradiotherapy and better locoregional control. In glioblastoma, RSI has been shown to independently predict overall survival, further supporting its cross-tumor-type applicability as a genomic marker of radiation response [100]. In a landmark clinical validation study, the Radiosensitivity Index demonstrated predictive value for tumor radiosensitivity, with radiosensitive tumors exhibiting significantly improved response and superior locoregional control following chemoradiotherapy (2-year control 86% vs. 61%) [101]. In breast cancer, RSI has demonstrated predictive value for tumor control, with radioresistant tumors exhibiting significantly higher local recurrence rates following radiotherapy, particularly within ER-negative and triple-negative subgroups [102]. More recent clinical analyses have extended these findings to additional disease sites. In endometrial cancer, RSI demonstrated clear predictive value for tumor control, with radioresistant tumors exhibiting significantly higher rates of pelvic failure and inferior pelvic control following radiotherapy, while no association was observed in patients not treated with radiation, confirming its role as a radiotherapy-specific biomarker [103]. Importantly, RSI has demonstrated characteristics of a predictive, rather than purely prognostic, biomarker. Its association with clinical outcome is predominantly observed in patients receiving radiotherapy, indicating that it reflects tumor-specific radiation sensitivity rather than general tumor aggressiveness. This distinction is critical for its potential application in treatment selection and personalization. In glioblastoma, RSI has also been shown to independently predict overall survival in patients treated with radiotherapy, with radiosensitive tumors demonstrating significantly improved survival outcomes (HR ≈ 1.64), particularly in methylguanine-DNA methyltransferase-unmethylated tumors [100]. Despite its promise, challenges remain regarding assay standardization, inter-platform variability, and prospective validation in randomized clinical trials. In addition, recent analyses have questioned its utility for direct dose-adjustment strategies when used in isolation, highlighting the need for integration with clinical and dosimetric parameters [104]. Nevertheless, RSI represents a foundational step toward biologically guided radiotherapy, providing a quantitative framework that links tumor genomics to radiation response and enabling the development of integrated dose personalization models.

Beyond gene expression-based predictors such as RSI, emerging approaches integrate genomic information directly with radiotherapy dose. The Genomic Adjusted Radiation Dose (GARD) model combines RSI with radiobiological modeling to estimate the biological effect of radiation in individual tumors, representing a step toward genomically guided dose personalization. In a pooled pan-cancer analysis of 1615 patients across multiple tumor types, GARD was found to be significantly associated with both time to recurrence and overall survival in radiotherapy-treated cohorts, whereas physical radiation dose alone was not predictive of outcome, supporting its role as a radiotherapy-specific biomarker and a potential framework for biologically individualized dose prescription [105].

Notwithstanding these encouraging results, molecular predictors of tumor radiosensitivity also face substantial barriers to clinical translation. An important limitation of many current genomic radiosensitivity approaches is that they are typically derived from pretreatment tumor biopsies and therefore may not fully capture the dynamic biological changes induced by radiotherapy itself. Radiation exposure can alter gene expression programs, DNA damage response signaling, immune activation, hypoxia, and clonal composition during treatment, potentially modifying tumor radiosensitivity over time. In addition, intratumoral heterogeneity and adaptive transcriptional responses may further limit the predictive stability of static baseline signatures. These considerations support the future development of longitudinal and adaptive biomarker strategies, including serial liquid biopsies, functional imaging, and dynamic molecular profiling during radiotherapy. Gene expression signatures frequently demonstrate reduced reproducibility across platforms, cohorts, and tumor types, while intratumoral heterogeneity and dynamic treatment-induced transcriptional changes complicate interpretation. Furthermore, many predictive models are derived from retrospective datasets and remain insufficiently validated in prospective randomized clinical trials. The integration of genomic signatures into radiotherapy decision-making additionally requires harmonization with established clinical and dosimetric parameters, as biological predictors alone may not adequately capture treatment response. These challenges highlight the need for integrated and clinically interpretable frameworks capable of combining molecular, functional, and clinical dimensions of radiosensitivity.

**Table 2:** Molecular determinants and predictive models of tumor radiosensitivity in precision radiotherapy.

Assay	Method	Results on the Patient	Cancer Type(s)	Reference
Clonogenic radiosensitivity (SF2)	Primary tumor cells isolated from pre-treatment biopsies and cultured in vitro, clonogenic survival assessed after irradiation (typically 2 Gy) by quantifying colony-forming ability (≥50 cells per colony).	Lower SF2 values were significantly associated with improved local control and overall survival, indicating that intrinsically radiosensitive tumors respond better to radiotherapy.	Cervical.	[87]
	Tumor biopsies processed to establish primary cell cultures; surviving fraction at 2 Gy determined using standardized clonogenic assays with colony counting after incubation	SF2 identified as an independent predictor of local tumor control with higher SF2 (radioresistant phenotype) correlated with increased risk of local recurrence following radiotherapy	Head-and-neck	[88]
	Soft-agar clonogenic assay using tumor-derived cells embedded in semi-solid medium to assess anchorage-independent colony formation after irradiation.	No significant correlation between SF2 and clinical outcome, suggesting limited reproducibility and highlighting methodological and biological variability.	Head-and-neck.	[89]
Tumor hypoxia and hypoxia gene signatures	Gene expression profiling of pre-treatment tumor biopsies using a hypoxia metagene derived from head-and-neck cancer and applied across tumor types to quantify tumor hypoxia status.	High hypoxia metagene expression was associated with increased tumor hypoxia and poorer response to radiotherapy, supporting its role as a predictive biomarker of radioresistance and potential selection tool for hypoxia-modifying or dose-escalation strategies.	Head-and-neck, multiple tumor types (validation cohorts depending on study).	[94]
	Gene expression profiling of pre-treatment tumor biopsies to develop and apply a 15-gene hypoxia classifier which stratified tumors into “more hypoxic” and “less hypoxic” groups.	Tumors classified as hypoxic were associated with significantly poorer clinical outcome following radiotherapy. The addition of hypoxia-modifying treatment improved outcomes in these patients, indicating that the signature has both prognostic and predictive value for tumor control.	Head-and-neck squamous cell carcinoma	[95]
	Gene expression analysis was performed on pre-treatment tumor samples using a predefined 26-gene hypoxia signature quantified by RT-qPCR (TaqMan low-density arrays). Patients were stratified into hypoxic (high score) and non-hypoxic (low score) groups and analyzed within randomized clinical trials of radiotherapy with or without hypoxia-modifying treatment (carbogen and nicotinamide).	In laryngeal cancer, hypoxic tumors (high signature score) showed significantly improved regional tumor control when treated with hypoxia-modifying therapy compared with radiotherapy alone (5-year control 100% vs. 81%). No predictive value was observed in bladder cancer, indicating tumor-type specificity of the biomarker.	Laryngeal and bladder.	[96]
Radiosensitivity Index (RSI)	Gene expression profiling of pre-treatment tumor samples using a 10-gene signature to generate a radiosensitivity score, applied to patients treated with concurrent chemoradiotherapy.	Lower RSI (radiosensitive tumors) was associated with significantly improved tumor response and higher locoregional control, with 2-year locoregional control rates of 86% versus 61% in radioresistant tumors, demonstrating predictive value for radiotherapy efficacy.	Head-and-neck, rectal, esophageal.	[101]
	Gene expression profiling of breast cancer tumor samples using a 10-gene RSI signature, applied to patients treated with breast-conserving therapy and radiotherapy to evaluate association with local recurrence risk.	RSI was associated with local recurrence risk following radiotherapy, with radioresistant tumors demonstrating higher rates of local failure. The predictive value of RSI was particularly evident in ER-negative and triple-negative subgroups, where RSI significantly stratified patients according to recurrence risk, supporting its role as a radiotherapy-specific biomarker.	Breast.	[102]
	Gene expression profiling of tumor samples using a 10-gene RSI signature applied to endometrial cancer patients treated with adjuvant radiotherapy to evaluate association with pelvic failure.	Radioresistant tumors (high RSI) demonstrated significantly higher rates of pelvic failure and worse pelvic control following radiotherapy (3-year pelvic control 84% vs. 100%). RSI was predictive of outcome only in radiotherapy-treated patients, supporting its role as a radiotherapy-specific biomarker.	Endometrial .	[103]
	Gene expression profiling of tumor samples using a 10-gene RSI signature applied to a TCGA glioblastoma cohort treated with radiotherapy ± temozolomide.	RSI independently predicted overall survival following radiotherapy; radiosensitive tumors demonstrated significantly improved survival (HR ≈ 1.64), with marked benefit in MGMT-unmethylated patients (1-year OS 84% vs. 54%).	Glioblastoma.	[100]

## 4. A Conceptual Research Framework for Integrating Functional, Genomic, and Clinical Radiosensitivity Data

To facilitate translation of radiosensitivity into clinically measurable parameters, we developed a conceptual mapping between model components and experimentally accessible variables (Supplementary File, Table S1), linking genomic, functional, and dosimetric features within a unified framework. Radiotherapy outcomes are governed by two fundamentally distinct but interdependent processes: tumor radiosensitivity, which determines TCP, and normal tissue radiosensitivity, which governs normal tissue complication probability NTCP [106,107]. Existing models, including RSI and GARD, primarily address tumor-intrinsic radiosensitivity, while patient-specific toxicity remains insufficiently integrated into predictive frameworks. One of the principal barriers to clinical implementation of radiosensitivity modeling has been the difficulty of integrating biologically heterogeneous variables into unified predictive frameworks. Functional assays, genomic signatures, imaging-derived parameters, and physical dose metrics differ fundamentally in dimensionality, scale, temporal behavior, and biological interpretation. Historically, this heterogeneity has limited reproducibility, model interpretability, and external validation, while also increasing the risk of overfitting in high-dimensional predictive models. These challenges are further compounded by variability in assay standardization, differences in treatment protocols, and limited availability of large prospective datasets containing harmonized biological and clinical annotations. To address this gap, we propose a conceptual unified biophysical systems model in which both tumor and normal tissue responses are described within a common formalism, enabling estimation of the therapeutic ratio (TR). Both tumor and normal tissues follow the linear–quadratic (LQ) model, but with different α and β determinants: SF = e^−(αD+βD2)^ [108]. For the tumor radiosensitivity module (T_resp_) we define α_tumor_ = a⋅M_RSI_⋅C_d_⋅M_hyp_ ⋅It, where a is the classical linear–quadratic radiosensitivity coefficient (Gy^−1^), M_RSI_ is genomic radiosensitivity (RSI-derived), C_d_ is damage complexity (related to LET and track structure) and M_hyp_ and It represent the tumor microenvironment (including hypoxia and immune activation). Thus, the tumor radiosensitivity module is a function of α_tumor_, β and dose D: T_resp_ = f(α_tumor_,β,D). The proposed expression for tumor response (T_resp_) should not be interpreted as a formal Poisson-based tumor control probability (TCP) model. Rather, it represents a conceptual surrogate reflecting radiation-induced tumor cell kill under linear–quadratic assumptions. For the normal tissue radiosensitivity module (NTCP) we define α_normal_ = S_g_⋅C_d_⋅M_normal_, where S_g_ is a patient-specific radiosensitivity factor associated with germline variants (e.g., ATM, XRCC1, TGFB1) and clinical radiosensitivity phenotypes, C_d_ represents radiation quality (shared between tumor and normal tissues), and M_normal_ represents the tissue-specific modifiers related to inflammation, vascular injury and fibrosis signaling. Thus, the normal tissue radiosensitivity module (NTCP) is a function of α_normal_, β_normal_, dose D, and the irradiated volume V: NTCP = f(a_normal_,D,V). The TR is defined as the balance between T_resp_ and NTCP: TR = T_resp_⋅(1 − NTCP). This balance represents the central objective of radiotherapy, aiming to maximize tumor control while minimizing toxicity. The proposed framework establishes a systems-level representation of radiotherapy response by explicitly coupling tumor radiosensitivity and normal tissue toxicity through shared physical and biological determinants. Unlike existing models that focus exclusively on tumor genomics, this approach integrates radiation quality (LET), microenvironmental factors, and patient-specific susceptibility into a unified formulation. Importantly, many model components can, in principle, be informed by measurable or inferable clinical and molecular data, including gene expression profiles (RSI), treatment planning-derived LET distributions, patient-specific radiosensitivity germline genetic variants through profiling of DNA repair and fibrosis-associated genes, and tumor hypoxia quantified using functional imaging modalities such as FMISO PET (see Supplementary Section S1 and Table S1). Although, their integration into a unified predictive system remains an area of ongoing research. Retrospective validation could be performed by correlating model-derived predictions of T_resp_ and NTCP with clinical outcomes in well-annotated radiotherapy cohorts. In particular, comparative studies between photon and proton therapy populations offer an opportunity to evaluate the contribution of LET-dependent damage complexity. Ultimately, this framework could support clinical decision-making by enabling biologically and physically informed treatment personalization, including selection of radiation modality, dose adaptation, and identification of patients at increased risk of toxicity. If prospectively validated, integrated radiosensitivity frameworks could eventually support clinically actionable radiotherapy adaptation strategies. Potential applications may include biologically guided dose escalation for radioresistant tumors, dose de-escalation in highly radiosensitive settings, individualized fractionation selection, modality selection (e.g., proton therapy or other high LET approaches in patients predicted to be at elevated risk of normal tissue toxicity or radioresistant disease), intensified monitoring or prophylactic interventions for patients at high risk of toxicity, and incorporation of hypoxia-targeted or radiosensitizing therapies. Such approaches could also contribute to refinement of T_resp_ and NTCP modeling within adaptive radiotherapy workflows. Representative interpretation examples of the conceptual TR framework are provided in Supplementary Table S2. However, these applications remain largely investigational, and substantial prospective validation will be required before integrated radiosensitivity-guided treatment modification can be routinely implemented in clinical practice. A major unresolved challenge in such integrative approaches is the heterogeneity of the contributing variables. Functional assays, genomic signatures, and physical dosimetric parameters differ fundamentally in dimensionality, scale, dynamic range, data structure, and biological interpretation, creating substantial challenges for integration, weighting, calibration, and interpretability. The present framework should not be interpreted as a validated predictive model, but rather as a conceptual systems-level roadmap intended to illustrate how heterogeneous biological, functional, genomic, and dosimetric variables could eventually be integrated within future precision radiotherapy strategies. To improve through conceptual transparency and mathematical rigor, we additionally introduce, in the Supplementary Materials, a preliminary quantitative approach, including representative equations describing integration principles and a calibration K-factor designed to account for scale heterogeneity and variable normalization across biological, genomic, and dosimetric domains.

Future implementation of clinically applicable integrated radiosensitivity models would require rigorous preprocessing and harmonization strategies, including normalization across variable types, biologically informed feature weighting, dimensionality-aware calibration, and robust validation against prospective clinical endpoints. At present, insufficient prospective data and lack of harmonized multi-modal datasets prevent robust clinical implementation of such unified models.

## 5. Discussion

Despite significant advances, no single biomarker or assay has yet achieved widespread clinical adoption. Functional assays such as RILA and γ-H2AX show promise for predicting normal tissue toxicity, but their implementation is limited by variability and lack of standardization. Similarly, genomic signatures and organoid-based models provide valuable insights into tumor radiosensitivity, yet require further validation in large, prospective clinical studies. The integration of functional, genomic, and clinical data remains a key challenge and represents a critical step toward clinically actionable predictive models in radiotherapy. Recent large-scale initiatives are beginning to translate radiosensitivity from a purely biological concept into a clinically actionable, systems-level phenotype. The emergence of large prospective initiatives such as PROSECCA highlights the growing recognition that robust multicenter validation and harmonized clinical datasets are essential prerequisites for translating radiosensitivity biomarkers into clinical practice. PROSECCA represents a prospective study protocol/cohort initiative designed to support future biomarker validation rather than a completed clinical validation study. In particular, this study links radiotherapy planning parameters, imaging features and longitudinal electronic health records from approximately 15,000 prostate cancer patients to develop artificial intelligence-based predictors of tumor control and normal tissue toxicity. By integrating >400 patient-specific variables with dosimetric and radiomic data, this multicenter study protocol aims to identify individuals at increased risk of radiation injury and to adapt dose and fractionation accordingly, with the long-term aim of supporting personalized radiosensitivity-informed care. Such population-scale modelling complements molecular biomarkers and suggests that future radiosensitivity assessment will likely require hybrid biological–clinical approaches rather than single-axis genomic tests [109]. The growing integration of artificial intelligence (AI) into radiotherapy research and clinical practice offers further opportunities through the incorporation of patient-reported outcomes (PROs), including systematic surveys capturing acute and late side effects experienced by patients. These real-world data provide a more comprehensive representation of treatment burden beyond clinician-graded toxicity scales. When integrated into AI-driven models alongside clinical, dosimetric, and molecular parameters, such data have the potential to improve the accuracy of toxicity prediction, enable dynamic risk stratification, and support personalized treatment adaptation [110,111]. However, challenges remain regarding data standardization, longitudinal collection, and integration across heterogeneous platforms. Beyond genomics, emerging multi-omics approaches, including proteomics, transcriptomics, metabolomics, and epigenomics, offer complementary insights into the molecular determinants of radiosensitivity by capturing dynamic functional states that are not fully reflected at the genomic level. Proteomic profiling, in particular, can provide direct information on protein expression, post-translational modifications, and signaling pathway activation, while metabolomic and transcriptomic data further characterize cellular stress responses and microenvironmental interactions. The integration of these high-dimensional datasets into AI-driven frameworks holds significant promise for advancing precision radiotherapy by enabling more accurate prediction of both tumor response and normal tissue toxicity. However, challenges related to data integration, standardization, and clinical validation remain substantial, and the translation of multi-omics-based models into routine clinical practice is still in its early stages [112,113]. In addition to functional and molecular assays, routine hematological parameters, particularly peripheral blood cell counts, represent a simple and clinically established means of monitoring treatment tolerance during radiotherapy. Radiation-induced leukopenia, reflecting depletion of circulating white blood cells and immune suppression, is a common dose-limiting toxicity, especially in patients receiving concurrent chemoradiotherapy. Dynamic changes in leukocyte counts during treatment can inform clinical decision-making, including dose modification, treatment interruption, or supportive interventions. While not a direct measure of intrinsic radiosensitivity, these parameters provide real-time insight into systemic treatment effects and may complement functional and molecular biomarkers within integrated predictive frameworks for personalized radiotherapy [114,115]. In parallel, liquid biopsy approaches are increasingly being evaluated as clinically feasible tools for monitoring tumor response to radiotherapy and informing treatment adaptation. Studies assessing circulating tumor DNA (ctDNA) dynamics during and after curative-intent radiotherapy have demonstrated that early reductions or clearance of ctDNA correlate with improved local control, relapse-free survival, and overall survival across several tumor sites, including lung, head-and-neck, and gastrointestinal malignancies [116,117]. Conversely, persistence or re-emergence of ctDNA following radiotherapy has been associated with molecular residual disease and early clinical relapse, often preceding radiographic progression by several months [118,119]. These findings suggest a potential role for liquid biopsy in identifying radioresistant disease during or shortly after treatment, enabling intensified surveillance or early therapeutic intervention. Importantly, liquid biopsy readouts primarily reflect tumor burden and clonal response rather than intrinsic normal tissue radiosensitivity. As such, ctDNA-based monitoring is best viewed as complementary to functional radiosensitivity assays and population-scale clinical models, together supporting adaptive, clinically actionable precision radiotherapy. Coordinated multicenter validation efforts, assay standardization, and harmonized computational frameworks will be essential to the establishment of reliable, actionable biomarkers capable of guiding toxicity risk stratification and individualized treatment adaptation in the next generation of precision radiotherapy.

In parallel with these emerging clinical and molecular monitoring strategies, functional radiosensitivity assays remain uniquely positioned to provide direct, patient-specific insight into the biological response to ionizing radiation. Unlike genomic or circulating biomarkers, which often infer radiosensitivity indirectly, functional platforms quantify the integrated cellular consequences of radiation exposure, including DNA repair capacity, chromosomal stability, apoptotic competence, and epithelial regenerative potential [53,54]. This phenotypic resolution is particularly relevant for normal tissue toxicity, where downstream biological responses rather than isolated molecular alterations ultimately determine clinical outcome [3,4]. As such, functional assays represent an essential complement to genomic and liquid biopsy approaches within a multi-dimensional framework of radiosensitivity assessment.

Despite substantial progress, several limitations continue to constrain the clinical translation of radiosensitivity biomarkers. A key limitation of current radiosensitivity research is that many biomarkers have been developed and validated in the context of radiotherapy alone, whereas most clinical protocols involve combined-modality treatments such as chemoradiation. This discrepancy may contribute to inconsistent predictive performance across studies and highlights the need for context-aware validation and integration of biomarkers within clinically relevant treatment settings. Functional assays are often limited by variability in experimental protocols, differences in cell source and culture conditions, and lack of inter-laboratory standardization, all of which affect reproducibility and scalability [43,71]. Similarly, genomic and transcriptomic biomarkers, while increasingly robust, frequently demonstrate modest effect sizes when considered in isolation and may not fully capture the dynamic and context-dependent nature of radiation response [8,10]. Liquid biopsy approaches, although highly promising for monitoring tumor dynamics, primarily reflect tumor burden and clonal evolution rather than the intrinsic radiosensitivity of normal tissues. Furthermore, many studies remain retrospective or exploratory, with a relative scarcity of large-scale prospective trials validating predictive performance across diverse patient populations and treatment settings. These limitations underscore the need for integrative models that combine complementary layers of biological and clinical information. Biological variability related to patient sex and age may further influence radiosensitivity and treatment outcomes. Differences in hormonal regulation, immune response, and tissue composition between males and females have been suggested to modulate radiation response, particularly in the context of inflammatory and fibrotic pathways. Similarly, age-related factors, including stem-cell regenerative capacity, tissue development, and long-term susceptibility to radiation-induced damage, are especially relevant in pediatric populations, where the risk of late toxicity and secondary malignancies is significantly higher. However, despite these observations, sex- and age-specific biomarkers of radiosensitivity remain poorly defined, and most functional and molecular assays have not been systematically validated across these subgroups.

Conceptual systems-level frameworks that explicitly integrate tumor control probability and normal tissue complication probability within a unified formalism offer a promising direction for advancing predictive radiotherapy. By linking model parameters to measurable biological, physical, and clinical variables (as outlined in Supplementary Section S1 and Table S1), such approaches provide a pathway toward translating multi-scale radiosensitivity into clinically actionable decision-support tools. Importantly, these models emphasize that radiosensitivity is not an isolated molecular property but an emergent phenotype arising from the interaction of genomic, microenvironmental, and treatment-related factors. Hybrid approaches incorporating germline genetic variation, functional radiosensitivity assays, tumor-specific molecular profiling, and real-time clinical or liquid biopsy data offer a more comprehensive representation of radiation response than any single modality alone. Within such frameworks, functional assays can anchor biological interpretation by providing direct phenotypic readouts, while genomic and transcriptomic data contextualize inherited and tumor-specific variability. Population-scale clinical modeling and artificial intelligence further enable the integration of these heterogeneous data streams into predictive tools that can be applied in routine clinical workflows [109]. Looking forward, the future of precision radiotherapy will likely depend on the development of standardized, scalable, and clinically validated multi-parameter models capable of guiding individualized treatment decisions. Advances in automation, high-content imaging, and computational analysis are expected to improve the reproducibility and throughput of functional assays, facilitating their integration into clinical pipelines. At the same time, increasing availability of large, well-annotated clinical datasets will support the refinement of predictive algorithms and enable prospective validation of combined biomarker strategies. Ultimately, the convergence of functional, genomic, and clinical modeling approaches has the potential to transform radiosensitivity from a descriptive biological concept into a quantitatively defined, clinically actionable parameter that can inform dose adaptation, toxicity mitigation, and treatment optimization.

## 6. Conclusions

Radiosensitivity is a multi-scale phenotype emerging from DNA damage complexity, cellular repair, tissue response, and systemic signaling. No single biomarker is sufficient for clinical implementation, and future progress depends on integrated, multi-dimensional models validated in prospective studies. Emerging integrative approaches combining functional, molecular, and clinical data offer a more robust framework for toxicity prediction and treatment personalization. Conceptual systems-level models that unify tumor radiosensitivity and normal tissue toxicity within a common predictive framework, supported by measurable biological and clinical parameters, represent an important step toward clinically actionable precision radiotherapy. Despite major advances in radiogenomics and functional radiosensitivity assessment, substantial challenges remain before biologically guided radiotherapy can be routinely implemented in clinical practice. Future progress will depend on improved assay standardization, prospective multicenter validation, harmonized toxicity endpoints, integration of multi-scale biological and dosimetric data, and the development of interpretable computational frameworks capable of robust external validation. Importantly, radiosensitivity should not be viewed as a static single-parameter property but rather as a dynamic systems-level phenotype influenced by tumor biology, host factors, treatment context, and temporal evolution during therapy. Addressing these challenges will be essential for translating radiosensitivity research into clinically actionable precision radiotherapy.

## Data Availability

No new data were created or analyzed in this study. Data sharing is not applicable to this article.

## Supplementary Materials

The following supporting information can be downloaded at https://www.mdpi.com/article/10.3390/cancers18111823/s1, Table S1: Mapping framework components to Measurable Clinical and Experimental Biomarkers; Table S2: Interpretation examples of the conceptual therapeutic ratio (TR) framework. References [29,45,77,86,90,95,96,97,120] are cited in the Supplementary Materials.
