# Supplementary material for: From Cellular Radiosensitivity to Precision Radiotherapy: Integrating Functional Assays, Genomics, and Clinical Modeling
Source: Cancers (Basel). 2026 Jun 2;18(11):1823. doi: 10.3390/cancers18111823 (PMC13256976; doi:10.3390/cancers18111823)
Supplement: Supplementary file 1 [file cancers-18-01823-s001.zip › cancers-4305401-supplementary.pdf]

cancers

MDPI

Supplementary data

From Cellular Radiosensitivity to Precision Radiotherapy: Integrating Functional Assays, Genomics, and Clinical Modeling

Angeliki Gkikoudi [1,2], Sotiria Triantopoulou [2], Eygenia Markellou [1], Vasiliki Xynou [2], Spyridon N. Vasilopoulos [1], Marios Myronakis [3], Evagelia C. Laiakis [4,5,6], Kiki Theodorou [3,7], Georgia I. Terzoudi [2] and Alexandros G. Georgakilas [1,*]

[1] DNA Damage Laboratory, Physics Department, School of Applied Mathematical and Physical Sciences, National Technical University of Athens (NTUA), Zografou Campus, 15780 Athens, Greece; angelikigkikoudi@mail.ntua.gr (A.G.); eygeniamarkellou891@gmail.com (E.M.); svasilopoulos@mail.ntua.gr (S.N.V.)
[2] Laboratory of Health Physics, Radiobiology & Cytogenetics, Institute of Nuclear & Radiological Sciences & Technology, Energy & Safety, National Centre for Scientific Research "Demokritos", 15341 Agia Paraskevi, Greece; iro@rrp.demokritos.gr (S.T.); vasiliki.ksn@gmail.com (V.X.); gterzoudi@rrp.demokritos.gr (G.I.T.)
[3] Department of Medical Physics, Faculty of Medicine, University of Thessaly, 41500 Larissa, Greece; myronakis@uth.gr (M.M.); ktheodor@med.uth.gr (K.T.)
[4] Department of Radiation Medicine, Georgetown University Medical Center, Washington, DC 20057, USA; ecl28@georgetown.edu
[5] Department of Oncology, Lombardi Comprehensive Cancer Center, Georgetown University Medical Center, Washington, DC 20057, USA
[6] Department of Biochemistry and Molecular & Cellular Biology, Georgetown University Medical Center, Washington, DC 20057, USA
[7] Innovation and Research, King Faisal Specialist Hospital & Research Centre, Riyadh 11211, Saudi Arabia
* Correspondence: alexg@mail.ntua.gr; Tel.: +30-210-772445

S1. Integrated Therapeutic Ratio (TR) Framework: Detailed Explanation

A conceptual systems-level framework integrating dosimetric, genomic, functional, and microenvironmental determinants of radiotherapy outcome is presented below. The equations are illustrative and intended to demonstrate model behavior rather than provide clinically validated predictions.

$$TR = T_{resp} \cdot (1\text{-}NTCP) = [1 - e^{-(a \cdot MRSI \cdot Cd \cdot Mhyp \cdot It \cdot D + \beta D^2)}] \cdot [e^{-(D \cdot V \cdot Sg \cdot Mnormal)/K)})] \quad (S1)$$

Where TR is the therapeutic Ratio (balance between tumor control and normal tissue toxicity), T_{resp} is a conceptual surrogate of TCP reflecting radiation-induced tumor cell kill under linear–quadratic assumptions, D the radiation dose, V the irradiated normal tissue volume , a is the classical linear–quadratic radiosensitivity coefficient (Gy^{-1}), M_{RSI} is a dimensionless radiosensitivity modifier, β the Sublethal damage/fractionation sensitivity, Cd the damage complexity modifier (LET/RBE effects), M_{hyp} a tumor hypoxia related component, It the Immune activation modifier, Sg the patient radiosensitivity integrating Radiation-Induced Lymphocyte Apoptosis (RILA), SNPs and γ-H2AX etc., M_{normal} the normal tissue modifier (fibrosis, inflammation, vascular injury) and K a scaling constant

 https://doi.org/10.3390/cancers18111823

used for normalization. The Patient Radiosensitivity Equation is described as:

$Sg = w_{RILA} \cdot R_{RILA} + w_{SNP} \cdot R_{SNP} + w_{\gamma\text{-}H2AX} \cdot R_{\gamma\text{-}H2AX} + \ldots +$. (S2)

The weighting coefficients (w) in Equation (S2) are intended to reflect the relative strength of evidence, reproducibility, and degree of clinical validation associated with each biomarker class rather than fixed quantitative contributions. Based on currently available studies, RILA and germline susceptibility markers, including SNP- and GWAS-derived signatures, may reasonably be assigned greater relative weights owing to their comparatively stronger prospective validation and reproducibility in predicting late normal-tissue toxicity.

To support the interpretability of the proposed conceptual framework, key components were qualitatively associated with representative biological, physical, and clinical variables. Table S1 provides an overview linking framework elements, such as genomic radiosensitivity (αRSI), radiation quality–dependent damage complexity (Cd), microenvironmental modifiers, and patient-specific radiosensitivity factors to experimentally accessible measurements. This mapping is intended to illustrate how multi-scale determinants of radiosensitivity may be related to measurable components, rather than to define a formalized or validated predictive model. The values presented are derived from heterogeneous literature sources and are included for conceptual illustration only.

Classical radiobiological components (α, β, α/β ratios)

The linear–quadratic formalism remains the dominant framework for describing radiation-induced cell killing, where the α component reflects irreparable (single-track) damage and β reflects accumulated sublethal damage. Jack Fowler demonstrated the clinical relevance of the α/β ratio in fractionation sensitivity, showing that early-responding tissues and many tumors exhibit high α/β (~10 Gy), whereas late-responding tissues show low α/β (~2–3 Gy), underpinning differential toxicity responses [86]. Eric J. Hall and Amato J. Giaccia further established that typical tumor α values fall in the range ~0.1–0.4 Gy^{-1}, with substantial variability across tumor types and experimental systems [45].

Genomic radiosensitivity indices (RSI / GARD)

The Radiosensitivity Index was developed by Javier F. Torres-Roca and

https://doi.org/10.3390/cancers18111823

Initial work showed that RSI is predictive of intrinsic radiosensitivity and correlates with survival after irradiation [97]. Subsequent clinical validations (breast, rectal, head and neck cancers) demonstrated that:

- Low RSI → improved locoregional control after radiotherapy
- High RSI → radioresistant phenotype

The extension into Genomic Adjusted Radiation Dose integrated RSI with the LQ model, showing in pooled analyses (>1,500 patients) that GARD correlates with clinical outcome, whereas physical dose alone does not. It should be noted that RSI values (~0.2–0.8) represent normalized gene-expression outputs, not physical components.

Damage complexity and radiation quality (C_d, LET, RBE)

Radiation quality is strongly linked to biological effectiveness through track structure and DNA damage clustering. Marco Durante and colleagues demonstrated that high-LET radiation (e.g., carbon ions) produces [120] dense ionization tracks, clustered DNA double-strand breaks and reduced repair fidelity compared with photons. For Relative biological effectiveness (RBE) values studies show:

- Photons: baseline (RBE ≈ 1)
- Protons: RBE ≈ 1.1–1.3 (variable with depth/LET)
- Carbon ions: RBE ≈ 2–5 depending on LET and tissue

These findings justify the use of C_d as a conceptual modifier, not a fixed component.

Germline susceptibility and functional radiosensitivity (S_g)

Inter-individual variability in normal-tissue toxicity has been linked to both functional assays and genetic variation. The RILA has been validated in multiple cohorts by multiple research groups which concluded that low RILA values are associated with increased risk of late fibrosis after breast radiotherapy [29]. In parallel, chromosomal radiosensitivity assays (e.g., G2 assay) show that increased chromosomal aberrations correlate with higher normal-tissue toxicity risk, while germline variants in DDR genes (e.g., *ATM*, *BRCA1/2*, *TGFB1*) associate with increased susceptibility to radiation-induced injury [77]. Together, these support Sg as a phenotypic conceptual modifier reflecting patient-specific radiosensitivity.

Microenvironmental modifiers (M_{hyp} and It and M_{normal}): Hypoxia and tissue response

https://doi.org/10.3390/cancers18111823

Tumor and normal-tissue microenvironments strongly modulate radiation response. Classical work by Louis Harold Gray established the oxygen effect and highlighted that oxygen enhances fixation of radiation-induced DNA damage [90]. The OER is typically ~2.5–3, meaning hypoxic cells are significantly more radioresistant. . The introduced parameter M_{hyp} does not directly represent the classical Oxygen Enhancement Ratio (OER). Rather, it was conceived as a normalized hypoxia-related weighting factor conceptually linked to oxygen-dependent modulation of radiosensitivity. Hypoxia gene signatures that hypoxic tumors have worse radiotherapy outcomes and benefit from hypoxia-modifying treatments, when in normal tissues Inflammation, vascular injury, and TGF-β–driven fibrosis pathways contribute to late toxicity and tissue remodeling [95,96].

Table S1. Mapping framework components to Measurable Clinical and Experimental Biomarkers

**Framework Component**	**Symbol**	**Biological Meaning**	**Measurement Method**	**Clinical Availability**	**Example Values**
Genomic radiosensitivity	M_{RSI}	Intrinsic tumor radiosensitivity (DDR, cell cycle, apoptosis)	Gene expression profiling (RSI panel), RNA-seq	Emerging (research / limited clinical)	0.15–0.4
Damage complexity (radiation quality)	C_d	DNA damage clustering driven by LET and track structure	Treatment planning systems, Monte Carlo simulations, LET maps	Available in proton/carbon centers	Photons ~1.0, Protons 1.2–1.5, Carbon 2–4
Tumor hypoxia	M_{hyp}	Oxygen-dependent radiosensitivity reduction	FMISO PET, HX4 PET, pimonidazole staining, HIF-1α IHC	Limited clinical use	0.3–1.0
Immune activation	It (Immune component)	Immune activation modifier.	RNA-seq (IFN signatures), cytokines, CD8+ T-cell infiltration	Research / translational	1.0–1.3
Patient radiosensitivity	S_g	Germline susceptibility to radiation toxicity	RILA, γH2AX and 53BP1 foci measurement, SNP analysis (*ATM*, *XRCC1*, *TGFB1*), Genome wide associated study (GWAS) panels	Limited but growing	1.0–2.5
Normal tissue microenvironment	M_{normal}	Fibrosis, inflammation, vascular damage response	TGF-β levels, cytokines, imaging	Partial clinical use	1.0–1.8
Dose distribution	D	Physical radiation dose	Treatment planning system	Standard clinical	1–80 Gy
Volume effect	V	Irradiated organ volume (NTCP driver)	DVH (Dose-Volume Histogram)	Standard clinical	Organ-specific
Clinical outcome (tumor)	T_{resp}	Relative biological tumor responsiveness to irradiation	Imaging Response Evaluation Criteria in Solid Tumors	Standard	0–1

https://doi.org/10.3390/cancers18111823

			(RECIST), survival data		
Clinical outcome (toxicity)	NTCP	Normal tissue complication probability	CTCAE scoring, patient-reported outcomes	Standard	0–1

These ranges are provided for conceptual illustration only and should not be interpreted as validated quantitative inputs for predictive modeling or clinical decision-making. Substantial variability exists across tissues, tumor types, and experimental systems, and formal component calibration requires prospective validation in well-annotated clinical datasets. To facilitate interpretation of the proposed conceptual therapeutic ratio (TR) framework, Table S2 provides illustrative clinical scenarios spanning a range of tumor response and toxicity conditions. These examples demonstrate how variations in T_{resp} and NTCP jointly influence the resulting TR score and the inferred therapeutic benefit.

Table S2. Interpretation examples of the conceptual therapeutic ratio (TR) framework.

**Example scenario**	**Tresp (Tumor response index) [0-1]**	**NTCP (Probability of complication) [0-1]**	**(1 - NTCP) (Tissue preservation factor) [0-1]**	**TR = Tresp (1 - NTCP) (Therapeutic ratio) [0-1]**	**Interpretation**
1. Optimal response	0.90 (High)	0.10 (Low)	0.90	0.81	High tumor response with low toxicity → High therapeutic benefit.
2. High response, high toxicity	0.90 (High)	0.70 (High)	0.30	0.27	High tumor response but unacceptable toxicity → Reduced overall benefit.
3. Low response, low toxicity	0.30 (Low)	0.10 (Low)	0.90	0.27	Low tumor response despite low toxicity → Low therapeutic benefit.
4. Moderate response, moderate toxicity	0.60 (Moderate)	0.50 (Moderate)	0.50	0.30	Moderate tumor response and toxicity → Moderate therapeutic benefit.
5. Poor response, high toxicity	0.20 (Very low)	0.80 (Very high)	0.20	0.04	Poor tumor response and high toxicity → Very low therapeutic benefit.
6. Effective response, near tolerance limits	0.80 (High)	0.40 (Moderate-high)	0.60	0.48	Good tumor response with toxicity near acceptable limits → Intermediate benefit

https://doi.org/10.3390/cancers18111823
